# Transcutaneous Vagus Nerve Stimulation Effects on Flavor‐Evoked Electroencephalogram and Eye‐Blink Rate

**DOI:** 10.1002/brb3.70355

**Published:** 2025-03-13

**Authors:** Samet Albayrak, Berfin Aydin, Gizem Özen, Faruk Yalçin, Merve Balık, Hüseyin Yanık, Burcu A. Urgen, Maria Geraldine Veldhuizen

**Affiliations:** ^1^ Cognitive Science, Informatics Institute Middle East Technical University Ankara Türkiye; ^2^ Department of Psychology and Neuroscience Bilkent University Ankara Türkiye; ^3^ Department of Psychology Penn State University University Park Pennsylvania USA; ^4^ Psychology Department Ludwig‐Maximilians‐Universität München Munich Germany; ^5^ Information Systems and Technologies Mersin University Mersin Türkiye; ^6^ Aysel Sabuncu Brain Research Center Bilkent University Ankara Türkiye; ^7^ National Magnetic Resonance Research Center (UMRAM) Bilkent University Ankara Türkiye; ^8^ Department of Psychology, Faculty of Humanities and Social Sciences Mersin University Mersin Türkiye; ^9^ Department of Anatomy, Faculty of Medicine Mersin University Mersin Türkiye

**Keywords:** EEG, flavor perception, multisensory perception, neuromodulation, vagus nerve

## Abstract

**Introduction:**

Chemosensory food signals are carried by the vagus nerve (VN) from the gut to the brain and these signals contribute to communicating fullness and caloric value of the consumed food in regulatory and reward‐related contexts. Here, we aimed to explore whether neural responses to flavor can be modulated through noninvasive VN stimulation, which can be done transcutaneously (transcutaneous vagus nerve stimulation [tVNS]) on the outer ear via the auricular branch of VN. The ideal stimulation location on the outer ear for tVNS is not agreed on but two candidate locations are cymba conchae and tragus.

**Methods:**

In this study, we explore the optimal stimulation location for tVNS (cymba conchae, tragus, and cymba conchae and tragus) and timing of tVNS relative to chocolate milk presentation (during, after) in a within‐participants design (15 participants). We examined various measures of efficacy; event‐related potential from electroencephalogram, eye‐blink rate, perceptual and hedonic aspects of flavor, swallowing behavior, and consumption behavior.

**Results:**

We observed no effect of stimulation location on any of the dependent variables. Unexpectedly, we observed a large effect of food consumption on spontaneous eye‐blink rate.

**Conclusion:**

In conclusion, overall we did not observe a clear optimal ear location for tVNS‐induced modulation of neurophysiological, perceptual, and behavioral variables. Future studies may confirm whether spontaneous eye‐blink rate can be a sensitive proxy for food reward‐related phasic dopamine shifts.

## Graphical abstract

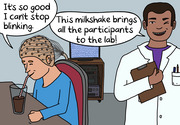

## 1 Introduction

The chemical senses play an important role in perceiving stimuli in our environment. This may range from identifying ingredients to determine if they are nutritious or harmful, to assessing perceptual aspects like intensity to decide to add more salt to food, to stating preferences to get a good recommendation from a restaurant app that uses artificial intelligence. Flavor perception is the conscious result of the integration of multiple sensory signals, consisting of olfaction, gustation, somatosensory signals, and potentially other sensory signals like auditory (Lawless and Heymann 2010; Stevenson 2016; Mizrahi 2017). A flavor stimulus can first be perceived from a distance with orthonasal olfaction, then once a food is placed in the mouth gustation and somatosensation plays an important role and upon swallowing, retronasal olfaction comes into play. Retronasal olfaction comes from volatiles from food that are carried up to the nasal cavity on the swallow breath (Lagast et al. 2017). The role of retronasal olfaction i flavor perception is relatively poorly understood and many neuroscience techniques are challenged by the difficulty of presenting retronasal olfactory stimuli in a controlled manner. However, studying flavor perception as a multisensory phenomenon including retronasal olfaction is important for a more realistic and holistic approach to perception (Heil 2011; Marks et al. 2007; Gibson 1966; Fjaeldstad et al. 2016). Flavor perception in humans is characterized by strong influences from learning and previous experience. For example, congruent gustatory and olfactory compounds that have been experienced together many times, such as strawberry odor and sweet taste, show supra‐additivity in intensity perception (Marks et al. 2007; Delwiche and Heffelfinger 2005; Murphy et al. 1977; Murphy and Cain 1980; McBride 1993) and detection thresholds (Dalton et al. 2000). Familiar flavors are also associated with increased liking and preference (Yeomans et al. 2008; Mela 2001; Birch 1999). These behavioral expressions are all thought to be the result of previous pairing with sweet taste and/or the presence of calories (Yeomans 2012; Prescott and Stevenson 2015). This flavor nutrient conditioning is thought to take place at least partially beyond the mouth, as there are no known receptors specialized for calorie detection in the mouth, and as mice lacking sweet receptor expression will still express conditioned flavor preferences (Ren et al. 2010; Sclafani et al. 2015; Sclafani 2004).

While not consciously perceived, food ingredients also bind to chemosensory receptors in the gut. These include taste‐like chemoreceptors that detect sugar and fats and are connected to the regulatory regions in the brain (Ackroff et al. 2010; Kaelberer et al. 2018; Williams et al. 2016). The information gathered from these receptors plays a crucial role on the eating behavior and the formation of food preferences (Han et al. 2018; Tan et al. 2020). In addition to slower signals carried in blood metabolites, the vagus nerve (VN) contributes to this information exchange between gut and brain with a fast monosynaptic pathway (Kaelberer et al. 2018). It consists of both afferent and efferent paths and regulates hunger and appetite in addition to making it possible for the brain to oversee digestion and related metabolic activities (Berthoud 2008; de Lartigue 2016; Yuan and Silberstein 2016).

Consumption of food with sugar or fat content causes the activation of chemoreceptors in the gut. Chemogenetic and optogenic stimulation of the cell bodies of the VN fibers that carry those signal triggers, by way of the nucleus of the solitary tract (NST), parabrachial nucleus, and midbrain, a dopamine response in the dorsal striatum (Han et al. 2018; Tellez et al. 2016). Such stimulation paired with consumption of novel flavors also leads to changes in behavior, for example, conditioned place and flavor preferences (Han et al. 2018).

Chronic intake of high calorie food can cause a loss of plasticity and sensitivity in afferent VN (De Lartigue et al. 2012; Kentish and Page 2014). This may in turn trigger a need for overconsumption in order to achieve a similar experience or brain response (de Lartigue 2016). This reward deficiency hypothesis (Kenny 2011) for obesity predicts that desensitization from overconsumption of highly caloric foods forms a vicious cycle where an organism will need to consume more and more high caloric foods to feel satisfied. In the long term, this causes a caloric surfeit, which will lead to weight gain, and eventually obesity (De Lartigue et al. 2014), which is associated with a high‐risk profile for metabolic disease.

Epilepsy patients who received a neck implant to electrically stimulate the VN experienced not only a reduction in seizures, but also lost weight and have less desire to consume sweet foods (Burneo et al. 2002; Bodenlos et al. 2007). This may indicate that electrical stimulation of VN has a regulatory effect on the dopamine response to food. Thus, it has been proposed that vagus nerve stimulation (VNS) may help reversal of desensitization of VN toward high caloric food (Öztürk et al. 2020). If dopamine release is promoted by VNS and if VN signals are crucial for establishing food reward, then VNS may alter the experience of food consumption and may eventually be used for promoting healthier eating behavior.

VNS is invasive, with limited applicability, but there are other ways to stimulate the VN. Transcutaneous stimulation of the auricular branch of the VN in the ear (ABVNS) has a similar effect as invasive VNS on neural responses (Kaniusas et al. 2019a; Frangos and Komisaruk 2017; Kraus et al. 2013) and epileptic seizures (Beekwilder and Beems 2010; Shiozawa et al. 2014) and is safe and easy to use (Hagen et al. 2014; Redgrave et al. 2018). However, the ideal location for stimulation is not agreed on (Burger and Verkuil 2018; Badran et al. 2018a; Butt et al. 2020). Two candidate locations for VNS through the ABVN are cymba conchae and tragus. The cymba concha is 100% innervated by ABVN (Peuker and Filler 2002; but also see Badran et al. 2018a; Butt et al. 2020; Burger and Verkuil 2018) and not by other auricular nerves, while the tragus is innervated by the ABVN, the greater auricular nerve and the auriculotemporal nerve (Peuker and Filler 2002). This makes the cymba conchae a more ideal stimulation target for experimental studies and confounding influences or noise from stimulating other nerves can be excluded. However both locations have been used in various trials and therapeutic applications, and for clinical effectiveness it may be optimal to actually use both locations. Various studies have compared responses between cymba conchae and tragus (Yakunina et al. 2017; Machetanz et al. 2021; Borges et al. 2021) without conclusive answers, but others have taken a more pragmatic approach of stimulating both (Altınkaya et al. 2023).

Various mechanisms for the effects of transcutaneous vagus nerve stimulation (tVNS) have been proposed. In the case of ABVN stimulation, these effects are presumed to all take place through stimulation of afferent pathways that will then influence VN brain targets like peripheral VN modulators do, triggering improved body–brain feedback loops (Kaniusas et al. 2019a). One of the proposed indirect mechanisms through the brain is the locus coeruleus–norepinephrine system primarily thought to involve enhanced memory function and enhanced task performance through increased arousal (Colzato and Beste 2020) and effects through the dopamine system and its role in learning, reward, and motivation (Neuser et al. 2020). Often heart‐rate variability (HRV) is used as a proxy for vagal tone in tNVS studies to screen for optimal stimulation parameters, but note that this is not an accepted valid biomarker (Borges and Laborde 2024; Burger et al. 2020). If tVNS partially exerts its effects through the dopaminergic system, then spontaneous eye‐blink rate (SEBR) may be a candidate physiological proxy for tVNS effectiveness. Certainly in studies of food reward, a proxy of dopamine function may be particularly relevant, as dopamine release in dorsal striatum scales with experienced meal pleasantness (Small et al. 2003) and dopamine signaling is crucial in flavor nutrient conditioning (Sclafani et al. 2011). SEBR is thought to reflect central dopamine function (Jongkees and Colzato 2016; Groman et al. 2014, but also see van der Post et al. 2004; Dang et al. 2017). We are unaware of investigations looking at the effect of food consumption on SEBR or studies looking at the effects of tVNS on SEBR, with the exception of a small pilot study that observed no effects of tVNS on SEBR (Öztürk et al. 2020).

While flavor perception and the effect of VNS on neural responses to food are both mostly studied with functional magnetic resonance imaging (fMRI), electroencephalogram (EEG) can provide additional information. EEG has various advantages over fMRI. It provides data with higher temporal resolution, allows participants to be positioned in any other way than laying down, and is less costly. Higher temporal resolution provides insight on fast mechanisms that occur after a short period of time (of milliseconds) in regard to stimuli. This makes it possible to investigate the near‐instantaneous changes occurring in the brain due to food consumption with the activity causing changes in event‐related potentials (ERPs). Recent studies show that EEG is a reliable method in measuring neural responses to oral stimuli (Wallroth et al. 2018; Andersen et al. 2019, 2020, 2019, 2020). It was also shown that the EEG responses to various sweeteners were different even if they were rated as perceptually identical (Brouwer et al. 2017). If participants can sit upright this will emulate a more naturalistic setup for eating. It also makes it viable to study retronasal olfaction from postswallow breath (Lagast et al. 2017) via a procedure involving sipping and swallowing of the chocolate milk, to our awareness not studied with EEG as of yet. However, EEG comes with its own set of challenges that need to be addressed, such as potential systematic influences and noise from electrical stimulation and muscle activity.

Effects of tVNS have not been extensively studied with EEG. A systematic review by Gianlorenco et al. (2022) reported effects of tVNS on early (100–300 ms) to late (450–600 ms) ERP components and a trend to increased power spectrum activity in lower frequencies in relation to tVNS. Most tVNS EEG studies have focused on enhancing cognitive processing, with to our awareness only one study directly related to food reward processing. Obst et al. (2020) used an oddball paradigm with food images and object images under tVNS and sham stimulation and observed a main effect of tVNS (on the P1, P2, and N2 amplitudes of the ERP), but not an interaction with image type. They also observed a (negative) correlation between P2 amplitude and post‐test food intake and suggested that future studies may investigate interactions between food consumption and tVNS.

To date few studies have looked at effects of tVNS on behavioral, hedonic, physiological, and neural responses to food stimuli (Yar et al. 2024). These studies are characterized by a wide range of methods and findings. There are no studies that have established a direct link between tVNS and dopamine release and vagal activity in the gut. However, there are indications of influences of tVNS on food perception, preferences, neural, and physiological processes. For example, tVNS increases liking of low fat flavors (Öztürk et al. 2020), decreases motivation to consume a flavor before consumption (Altınkaya et al. 2023), increases willingness to work for wanted food reward (Neuser et al. 2020), increases liking for food images in participants with anhedonia in major depression disorder (Koepp et al. 2021), decreases liking for food images in healthy participants (Alicart et al. 2021), increases in milk intake in infants with oral feeding dysfunction (Badran et al. 2020; Jenkins et al. 2023), induces changes in gastric wave amplitude, gastric frequency, and gastrin release (Hong et al. 2019; Teckentrup et al. 2020), increases neural responses to liked food images in food reward regions (Alicart et al. 2021), and increases stomach–brain coupling signals (Müller et al. 2022a). There are also various studies that show no effects of tVNS on these outcomes (reviewed in Yar et al. 2024), so additional work on the effect of tVNS on food perception and consumption is needed.

Here, we explored the effect of stimulating various locations of the ABVN on neural, physiological, behavioral, and perceptual responses to flavor perception of food stimuli. Previous studies investigating neural responses to food stimuli have timed tVNS prior (Alicart et al. 2019; Müller et al. 2022b) or concurrent (Obst et al. 2020) with the presentation of food stimuli, but it is currently unknown which may be more effective. In this study, we explore the optimal stimulation location for tVNS (cymba conchae, tragus, ancymba conchae and tragus) and timing of tVNS relative to chocolate milk presentation (during, after). We examined various measures of efficacy; ERP, resting‐state EEG, perceptual and hedonic aspects of flavor, swallowing behavior, SEBR, and consumption behavior.

## 2 Methods

### 2.1 Participants

This study is an exploratory study and sample size was determined by available resources rather than a power analysis. Previous similar studies for tVNS location testing included 6–30 participants (Romoli et al. 2014; Hagen et al. 2014; Badran et al. 2018b; Fallgatter et al. 2003). We identified six tVNS studies that used perception of food stimuli (odors, flavors, food images; Maharjan et al. 2019, 2018, 2019, 2018; Öztürk et al. 2020; Altınkaya et al. 2023; Müller et al. 2022b; Alicart et al. 2019) and sample sizes ranged from 11 to 82, with a mean of 27.67 SD of 26.88, and a median of 19. In our previous study with a fairly similar design (within‐participants, ratings of chocolate puddings, acute tVNS) with 11 participants, we observed changes in liking as a result of tVNS with an effect size of 1.00 (Öztürk et al. 2020). We used this to post hoc calculate (with a desired power of 0.80 and alpha probability of 0.05), a desired sample size of 10 (G*Power 3.1.9.7). We do note that power analyses from small samples (11 participants in this case) tend to underestimate sample size needed (Albers and Lakens 2018).

Fifteen healthy participants (seven women, eight men), with average age of 28.8 ± 6.17 and mean body mass index (BMI) of 24.5 ± 3.86 kg/m^2^ participated in the study. All participants reported having no known taste, smell, neurological, eating, psychiatric (excluding depression and attention‐deficit/hyperactivity disorder [ADHD]), cardiological, metabolic, or other pathological disorders. Three participants had ADHD with mild symptoms and four participants reported a past depression. No participants were on medication for at least for 6 months during their participation. The data for this study were collected between May of 2021 and August of 2022. Participants were recruited by word of mouth from around campus by the research team. The Mersin University Committee for Clinical Research approved the study protocol (number 7807789/050.01.04/1152384) and written informed consent was obtained from all study participants. All participants reported having not had COVID‐19 and after the vaccines were available, most participants had been vaccinated with two doses.

### 2.2 Design and Session Procedure

The design of the experimental setup was constructed to accommodate the challenges of combining EEG with a sip‐and‐swallow procedure and electrical stimulation. A sip‐and‐swallow procedure would cause muscle activity and movement that might lead to excessive noise in the EEG recording. This was solved in part by minimizing the movement with a forehead rest. We also recorded muscle activity from the main muscle involved in swallowing. Another source of noise in the EEG recording may come from the electrical pulses from the tVNS device. We antisynchronized the flavor stimulus delivery with the on‐periods of the tVNS, such that data of interest are collected while the tVNS device is not actively sending electrical pulses to the participant's ear.

**FIGURE 1:**
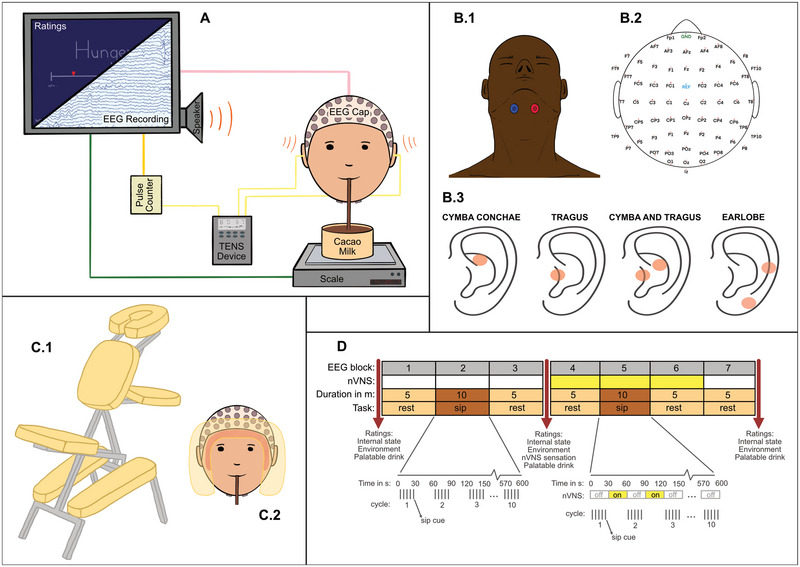
(A) Overview of experimental setup: Visual Analog Scales (VAS) were presented on a monitor for the participants to make perceptual ratings. Electrophysiological activity is obtained by electroencephalogram (EEG) electrodes on the cap and recorded on a separate computer. Participants sipped the chocolate milk through a straw from a bowl. The weight of the bowl was recorded by the USB‐readable scale in real time, thus sip size information was collected. The transcutaneous electrical nerve stimulator (TENS) device was connected to the electrodes on the participant's ears and also to the computer through a digital input/output device so sipping cues were never delivered at the same time as the on‐cycle of the TENS device. (B.1) Two electrodes were placed on submental muscle to identify the time point for peak muscle activity caused by swallowing. (B.2) Locations of 66 EEG electrodes (64 channels, one reference electrode, and one ground electrode). (B.3) Stimulation locations for all four conditions are shown. (C.1) The massage chair implemented to support an upright position. (C.2) Participant's head position showed on the headrest (from below) with lower jaw free to move without causing the whole head to move. (D) Seven blocks of EEG recording took place per session, identical for each stimulation location. During Blocks #1–3, and Block #7 no transcutaneous vagus nerve stimulation (tVNS) stimulation (yellow) was delivered. During Blocks #4–6 tVNS stimulation was delivered. Blocks #2 and 5 were sipping blocks (brown), others were resting blocks (light orange). Ratings (arrows) of internal and environment states, and the drink were presented before Block #1, between Blocks #3 and 4, and after Block #7. Ratings of tVNS sensations were requested only between Blocks #3 and 4. In sipping blocks, there were 10 cycles of five sips. Each cycle of five sips was 30 s long (visualized under Blocks #3 and #5), with sip cues evenly spaced. Sipping cycles were antisynchronized with the tVNS stimulation in Block #5, such that sipping occurred only during the “off” times, and never during the “on” times in the tVNS stimulation cycle.

We used a within‐participants design, with four sessions per participant. Each session took place on a different day and in each session tVNS was delivered to a different location: cymba conchae, tragus, lobe (sham), or tragus and cymba conchae (counterbalanced across participants; Figure 1B.3). We used four possible orders of conditions over sessions: sham–CT–C–T (order 1), CT–T–sham–C (order 2), T–C–CT–sham (order 3), and C–sham–T–CT (order 4). This follows the Williams design, a generalized variation of the Latin square design that is also balanced for first‐order carryover effects, and where carryover balance is achieved with very few subjects (Williams 1949). The number of participants assigned to these orders were: order 1, *n* = 4, order 2, *n* = 4, order 3, *n* = 4, order 4, *n* = 3. Participants were scheduled for the same time of day for each session. The session was completed within approximately 120 min (including prerecording EEG procedures such as fitting the cap and filling the electrodes with gel) for each participant. The four sessions for each participant were usually completed within a week or 2. The participant was asked to be neither too full nor too hungry during the sessions, so they were instructed to eat a light meal 2 h before the start of the session. They were also asked not to smoke or drink coffee on the day of a session and not to consume alcohol the day before. These were for both the metabolic stability of the participants and the sake of clean, higher quality EEG recordings.

Each session was identical in terms of their timeline and the events. The only difference between sessions was the location in the ear for tVNS. All EEG recordings and other experimental steps such as ratings were performed in a thermoneutral and quiet room. Participants were asked to use the bathroom and empty their bladder before the session began. Then they were outfitted with the cap and electrodes for EEG and electromyogram (EMG) recording and electrodes for tVNS stimulation. Then we adjusted stimulation intensity for each participant individually (see tVNS section below for details). Then the participants were moved from the preparation room to the chair in the recording room. The chair we used is different from the type of desk chair that is usually used for EEG studies. We used a massage chair that supports the shins, the chest and forehead. We selected this type of chair, because it has a horseshoe shaped headrest, that allows the lower jaw and mouth area to move freely, while supporting the forehead. This makes it possible for participants to sit still without needing to engage head and neck muscles, while allowing for swallowing (Figure 1C.1,C.2). The timeline of the experiment is shown in Figure 1D. When the participant was comfortable in the chair, we asked them to make ratings of their perception of their internal and environmental states as well as the evaluation of the chocolate milk. We refer to this first set of ratings as “Before Block #1, tVNS off.” Then we instructed the participants to look at the provided fixation cross and not to close their eyes or sleep, and commenced the EEG recording. For the first three blocks of EEG recording, the tVNS electrodes were in place, but the stimulation was kept turned off. The first block (“Block #1, rest, tVNS off”) was a 5‐min resting‐state EEG recording during which the participant looked at a fixation cross. Then the chocolate milk was brought into the recording room and placed on the scale in front of the participant and we started a 10‐min sipping block, “Block #2, sipping, tVNS off.” After Block #2, the chocolate milk was removed and Block #3 (“Block #3, rest, tVNS off”) was started, during which the participant looked at the fixation cross for 5 min. After Block #3 ended, we turned on the transcutaneous electrical nerve stimulator (TENS) device for tVNS delivery. The participant again rated internal and environmental states, the chocolate milk, as well as sensations of the tVNS stimulation, we refer to these ratings as “Before Block #4, tVNS on.” After they finished this set of ratings, they continued with block #4 which was another 5‐min resting‐state block with the participant looking at the fixation cross, “Block #4, rest, tVNS on.” Then the chocolate milk was brought back to the room and we started another 10‐min sipping block, “Block #5, sipping, tNVS on.” After the chocolate milk was removed from the recording room once more another 5‐min block was started, “Block #6, resting, tVNS on.” Between Blocks #6 and #7, the TENS device was turned off. The last block (“Block #7, rest, tVNS off”) started and took 5 min to complete. During both the last two resting blocks (Blocks #6 and #7) the participant looked at the fixation cross. Then we stopped the EEG recording and the participant rated their internal and environmental states, and the chocolate milk once more, “After Block #7, tVNS off.”

This timeline was designed to evaluate two types of neural responses. First, we wanted to examine ERPs to sipping of the chocolate milk before and during tVNS. Second, we wanted to examine neural oscillations before, during and after tVNS, as well as before and after sipping blocks.

At the end of the fourth session, participants were given a questionnaire about their awareness of the experimental manipulations before they were debriefed about it.

### 2.3 tVNS

Mild electrical stimulation was delivered to the various locations on participants’ ABVN through the skin of their ears. Tragus, cymba, and cymba and tragus were the ad verum experimental conditions, in addition to the earlobe as a sham/control condition. The conditions were each applied in separate sessions in a counterbalanced order. We used a TENS (Twin Stim Plus 3rd edition, Roscoe Medical Inc., Middleburg Heights, Ohio, USA) device. Both ears were stimulated for all sessions/conditions and for each location a differently shaped electrode was used. Electrodes for sham were two one‐sided clips that were placed on the earlobe and helix where there is no VN innervation. For the Cymba condition, a clamp that contacts the cymba concha from the anterior and posterior side of the outer ear was used. Electrodes for the tragus condition were two‐sided clips that were attached to the tragus. For the tragus‐and‐cymba condition two‐sided electrodes were placed in the ear to contact the inner sides of both of the locations of interest simultaneously. Figure 1B.3 shows the stimulation locations. The electrodes were taped into place, which minimized changes in the placement of the electrodes throughout the session and also minimized drying out of the electrode gel. By reducing both these factors, we also reduced changes in skin conductance and electrode positioning.

While most participants were blinded to sham/control versus active stimulation locations, three of the participants were part of the research team and they were aware of the conditions. Parameters for tVNS were same throughout the sessions and conditions and were as follows: a biphasic square wave pulse at 25 Hz and a pulse width of 250 µs, with a duty cycle of 30 s on, 30 s off. The current we deliver is biphasic, meaning the anode and cathode alternate constantly. These parameters are most commonly used in the literature and very similar to those used by the Nemos device (Thompson et al. 2021; Yap et al. 2020). The total stimulation duration was approximately 20 min that corresponds to the fourth, fifth, and sixth blocks of EEG recording as shown in Figure 1D.

Stimulation voltage was constant throughout the experiment. Amplitude was adjusted with a commonly used protocol (Farmer et al. 2021; Kaniusas et al. 2019b) for each participant and for each session by incrementally increasing the amplitude to the highest possible point without causing any pain or discomfort. With the help of this calibration process, it was ensured that the sensation across conditions stayed similar and the perception‐related placebo effects were avoided despite the anatomical differences between stimulation locations. The adjustment procedure was conducted as follows: after the electrodes were placed, the intensity was incrementally increased and participants were asked to report any pricking, burning, or stinging sensation to identify their pain threshold. Then the intensity was incrementally decreased to a point where participants experienced a tingling, vibrating sensation without pain and discomfort. The intensity was kept constant throughout the session unless the participant reported discomfort. In that case, intensity was readjusted and recalibrated to a comfortable level. No adverse effects were observed.

We used a digital input/output device (National Instruments USB‐6501, Austin, Texas, USA) connected to one of the TENS channels to determine when the tVNS cycle was on, so we could antisynchronize the sipping cues to the tVNS stimulation. Thus, the 30 s periods that contain sipping cues never overlapped with the electrical pulses delivered to the ear. MATLAB code to read the tVNS signal can be found here: https://github.com/mariaveldhuizen/TENS‐gated‐matlab‐trigger

### 2.4 EEG Recordings

High‐resolution EEG was recorded using 64 active electrodes, locations shown in Figure 1B.2 (Brain Products actiCAP active electrode system Version II, Brain Products GmbH, Germany). The Brainvision recorder (Brain Products GmbH, Germany) was used as recording software. Participants were prepared for recording in the setup room, adjacent to the recording room, by having the cap and EMG electrodes placed on them. Submental muscles that affect swallowing contain the digastric, the mylohyoid, and the geniohyoid muscle (Wheeler et al. 2007). To record swallowing‐related electrical signals, we placed the two EMG electrodes that were included in the EEG recording equipment and normally used for electro‐oculography on the submental muscles. The EMG electrodes were placed under the participant's jaw on the submental muscle as shown in Figure 1B.1, for detection of swallowing. A reference electrode for EMG electrodes was placed on the back of the participant's neck. All the electrodes were checked and optimized (via actiCAP ControlSoftware, Brain Products GmbH, Germany) in terms of impedance (less than 20 k*ohm*) and signal quality before moving the participant to the recording room. Ground and reference electrodes for EEG were placed on the Fpz and FCz locations, respectively. The sampling rate was 1000 Hz.

We wrote a MATLAB (R2020a) script to send markers via a parallel port connection to the EEG recording computer to mark the beginning and end of each block as well as the time of auditory sipping cue in the EEG recording. This script also played the auditory cues (Windows native default beep sound “beep.wav”) to cue sips. It also contained code to antisynchronize the sip cues from the tVNS delivery. This was achieved with the help of the digital input/output device that was used for connecting the TENS device to the computer. Additionally, the script queried an electronic scale (Ohaus NV622 EU, Parsippany, New Jersey, USA) connected by USB to the computer 50 times during the 6 s postsip cue to track consumption of the chocolate milk.

### 2.5 Food Stimulus

The food stimulus was chocolate milk, generally perceived as palatable, made from commercially available ingredients. The milk was prepared with 10% fat and 10% sugar weight‐by‐weight (w/w). The ingredients used were 3.0% fat shelf‐stable milk (SEK), 35% fat shelf‐stable cream (İçim), white sugar (Balküpü), vanilla sugar (Dr. Oetker), cacao powder (Dr. Oetker Gourmet Cacao). We mixed the chocolate milk 1 day before the test day and kept it in the refrigerator after waiting for it to cool to room temperature (see Supporting Information for the recipe). On the test day, the chocolate milk was taken out of the refrigerator, stirred, and brought to room temperature, before being served to the participant in a container with a flexible straw. We placed a 300 mL bowl on the electronic scale, filled more than halfway at the beginning of each sipping block (Blocks #2 and #5). Between these two blocks, more of the chocolate milk was added to make sure that the bowl would not empty during the block. Each participant consumed approximately 300–400 mL of chocolate milk every session.

### 2.6 Behavioral Ratings

#### 2.6.1 Internal and Other States Ratings

A horizontal 101‐point Visual Analog Scales (VAS) used for participants to report their internal states and perception of environmental conditions. VAS were presented on a monitor with a dark background and participants selected the desired value by dragging the cursor on a line. Ratings were made in this order: hunger, fullness, thirst, need to pee, stress, comfort, sleepiness, tiredness, and temperature. All states but temperature were on a unipolar 0–100 VAS with “not … at all” versus “extremely ….” VAS for temperature was bipolar from −50 to 50, labeled with “very cold” on −50, “good” on 0, and “very hot” on 50. Instructions and explanations on these ratings were given to the participants during the first session they attended. These ratings were collected to identify any potential confounding variable, but also to monitor participants’ levels of comfort and discomfort during the session and make adjustments if necessary.

#### 2.6.2 tVNS Stimulation Perception Ratings

Participants were also asked to rate their perception of sensations caused by tVNS on their ears. Unipolar VAS were used for; intensity, tingling, vibration, itching, stinging, and burning. The scales were labeled as “no sensation” and “most intense sensation” on each end. Bipolar VAS were used for pleasantness with labels “very unpleasant,” “neutral,” and “very pleasant.”

#### 2.6.3 Chocolate Milk Perception Ratings

After taking one sip from the chocolate milk and swallowing, participants were asked to rate the drink in these categories: intensity, liking, familiarity, sweetness, sourness, bitterness, creamy, fatty, other, caloricness, healthiness, and desire to consume more of the drink (referred as “wanting” from here on). Liking, familiarity, and healthiness were rated on a bipolar scale while intensity, sweetness, sourness, bitterness, creamy, fatty, caloricness, and “other” were rated on a unipolar scale. Presence of the “other” scale was aimed to give participants a way of reporting any other attributes that do not match with the rest of the scales such as chocolateness or presence of an off‐taste, to avoid any halo‐dumping effects (Lawless and Heymann 1999).

#### 2.6.4 Debriefing Questionnaire

We assessed effectiveness of blinding or awareness of the sham versus “active” tVNS locations with a structured questionnaire after completion of the experiment. The questions were organized in a progressive manner, such that we first asked the questions that addressed the least understanding and knowledge of the experimental design and VN, and the most sophisticated understanding and knowledge last. For example, we first asked if the participant thought a placebo condition was involved, then which that might be, then what they knew about the VN, then which condition was associated with VN stimulation, and so on. As measured by this debriefing questionnaire, four participants showed complete accurate awareness of the placebo and active conditions and could be classified as “not naïve.” Eight participants showed partial awareness or correct guesses of the placebo or active conditions and could be classified as “somewhat naïve,” and three participants showed no awareness of placebo nor of the active conditions and could be classified as “completely naïve.”

### 2.7 Data Processing

#### 2.7.1 EEG Data

Analysis of EEG data was done with the open source EEGLAB (Delorme and Makeig 2004) and ERPLAB (Lopez‐Calderon and Luck 2014) softwares. The statistical analysis with mass univariate analysis was done with FMUT (Fields 2017) on MATLAB, spatiotemporal cluster analysis was done in MNE (Gramfort 2013) on Python.

First, data were preprocessed including the filtering, re‐referencing, and epoching steps. Low‐pass and high‐pass filtering was done to obtain signal components below 50 Hz and above 1 Hz. The data were epoched time‐locked to the peak swallowing moment (from −2000 to 1999 ms), which was obtained via processing the EMG signal. Following the epoching, independent component analysis (ICA) was performed on each session's dataset to identify and prune signals occurring due to blinks and eye movements. Then we performed artifact rejection using the semiautomated procedures implemented in EEGLAB. This included improbable data (by joint probability) and abnormal trends (by kurtosis of activity) methods followed by visual inspection. After all the suitable epochs were extracted for each session, trials were grouped according to the experimental conditions they correspond to and averaged.

#### 2.7.2 Submental EMG Data

We used MATLAB 2019 for filtering and feature extraction. Obtained raw EMG signals were smoothed by removing the DC offset noise first. Then we used a fifth‐order Butterworth bandpass filter with the low and high cut‐off frequencies as 25 and 400 Hz. Finally, signals were passed through a notch filter to filter out the power network noise. To identify swallows, we applied a hybrid algorithm including the Teager–Kaiser energy operator (TKEO; Solnik et al. 2010) and discrete wavelet transform to improve signal‐to‐noise ratio and detect EMG bursts (Restrepo‐Agudelo et al. 2017). We then applied wavelet decomposition to make swallow patterns clearer using “symlet 4” as the mother wavelet. Finally, we calculated the root mean square to detect swallow characteristics and assess the power of the swallows in the decomposed signal (see Supporting Information Figure 1). For all blocks that cleaned signal was displayed over time with swallow cue markers for sipping blocks. For each block, the researcher Samet Albayrak selected a threshold that captured the visually identified swallow signals. If a swallow fell in a window of 4000 ms with the swallow cue onset = 0 ms, it was marked as a “cued swallow.” If there was more than one swallow in this interval, the first one was marked as a “cued swallow” and any subsequent swallows as a “spontaneous swallow.” Any swallows outside the 30 s cued sipping interval and in resting blocks were marked as “spontaneous swallows.” For each of the swallows, we calculated the following characteristics: peak latency relative to cue, peak amplitude, and area under the curve.

#### 2.7.3 EOG Signal From Frontal Channels for Eye‐Blink Rate

We extracted the signal from the frontal channels Fp1, Fp2, AF7, and AF8 for the detection of eye blinks. We used the BLINKER plugin for EEGLAB (Kleifges et al. 2017) to calculate the average eye‐blink count per minute (referred to as “eye‐blink rate”). The BLINKER algorithm first identifies potential blinks after bandpass filtering (1–20 Hz) and then marks intervals in which the signal is greater than 1.5 times the standard deviation above the overall signal mean. Then the algorithm excludes nonblink eye movements that do not meet the prototypical rounded “tent‐like” shape with a fitting procedure that calculates the correlation between the observed and prototypical blink. Then low signal‐to‐noise candidate signals and blinks are rejected. Lastly, the number of blinks in the channel of the recording with the most “good” blinks is calculated. We averaged eye‐blink rates over block durations of 5 min or more, which follows the recommendation of averaging over intervals of at least 3–5 min (Zaman and Doughty 1997). Note that this procedure may eliminate blinks that are contaminated by other eye movements, so it is not meaningful to interpret absolute eye‐blink rate in this case. Since we assume that eye movement other than blinks are unlikely to be influenced by tVNS, we reasoned that any observed differences between the eye‐blink rates may still be meaningful.

### 2.8 Statistical Analyses

Statistical analyses for all data except the ERP data were conducted with JASP 0.18.3. Jasp was also used for data visualizations. Planned follow‐up *t*‐tests were conducted to compare the effect of location, the effect of time or interaction effects. These were corrected for multiple comparisons using the Holm correction. Alpha was set at 0.05 here and in all following analyses. To reduce an inflated false positive rate from multiple *F*‐tests within an analysis of variance (ANOVA) and multiple *F*‐tests in a family of ANOVAs (Cramer et al. 2016), we implemented a false discovery rate (FDR) correction within each ANOVA and across families of tests (the family of VNS perception ratings, the family of drink perception ratings, the family of internal state ratings, and the family of swallowing parameters), and report these corrected values along with significant uncorrected *p*‐values.

For the VNS level selected individually for each participant, we conducted a repeated‐measures ANOVA with within‐subjects factors “location” with four levels (sham, cymba conchae “C,” tragus “T,” and cymba conchae and tragus “CT”) and side (right vs. left). To account for an inflated false positive rate for multiple tests within an ANOVA, we corrected for FDR with the Benjamini–Hochberg procedure for a family of three tests.

For each of the tVNS ratings, we conducted a repeated‐measures ANOVA with within‐subjects factor “location” with four levels (sham, cymba conchae “C,” tragus “T,” and cymba conchae and tragus “CT”). To account for an inflated false positive rate for multiple tests within an ANOVA, we corrected for FDR with the Benjamini–Hochberg procedure for a family of seven tests.

For the eye‐blink rate, we ran the following analysis to examine the effect of tVNS stimulation and food stimulation over time, we conducted a repeated‐measures ANOVA with within‐subjects factors block (1–7) and location (sham, cymba conchae “C,” tragus “T” and cymba conchae and tragus “CT”). To account for an inflated false positive rate for multiple tests within an ANOVA, we corrected for FDR with the Benjamini–Hochberg procedure for a family of three tests.

For each of the internal and environmental state ratings and drink perception ratings, we conducted a repeated‐measures ANOVA with within‐subjects factors “time” with three levels (“Before Block #1, tNVS off,” “Before Block #4, tVNS on” and “After Block #7, tVNS off”) and “location” with four levels (sham, cymba conchae “C,” tragus “T,” and cymba conchae and tragus “CT”). To account for an inflated false positive rate for multiple tests within an ANOVA, we corrected for FDR with the Benjamini–Hochberg procedure for a family of 27 tests for the internal and environmental state ratings, and for a family of 36 tests for the drink perception ratings.

With the electronic scale, we collected the weight of each sip during the sipping blocks. With these data, we conducted a repeated‐measures ANOVA with within‐subjects factors “time” with two levels (“Block #2, sipping, tVNS off” and “Block #5, sipping, tVNS on”) and “stimulation location” with four levels (sham, cymba conchae “C,” tragus “T,” and cymba conchae and tragus “CT”) and “cycle” with 10 levels (1–10) for each of the 10 sipping cycles of five sips. To account for an inflated false positive rate for multiple tests within an ANOVA, we corrected for FDR with the Benjamini–Hochberg procedure for a family of three tests.

For the EMG signal that we collected to measure swallowing, we specified a repeated‐measures ANOVA with within‐subjects factors “time” with two levels (“Block #2, sipping, tVNS off” and “Block #5, sipping, tVNS on”) and “stimulation location” with four levels (sham, cymba conchae “C,” tragus “T,” and cymba conchae and tragus “CT”). We ran this for both the peak amplitude and latency of peak amplitude of this signal. To account for an inflated false positive rate for multiple tests within an ANOVA, we corrected for FDR with the Benjamini–Hochberg procedure for a family of four tests.

The average ERP for each stimulation location and time point for each participant was entered into a within‐subjects 4 × 2 ANOVA with factors “time” with two levels (“Block #2, sipping, tVNS off” and “Block #5, sipping, tVNS on”) and “stimulation location” with four levels (sham, cymba conchae “C,” tragus “T,” and cymba conchae and tragus “CT”) with the spatiotemporal cluster analysis module in MNE (Gramfort 2013) in Python. Next, we used the Factorial Mass Univariate Toolbox (Fields 2017) in MATLAB to conduct *t*‐tests between stimulation locations (sham, cymba conchae “C,” tragus “T,” and cymba conchae and tragus “CT”) in “Block #5, sipping, tVNS on” only. We also conducted *t*‐tests for each stimulation location against 0 to examine which stimulation location shows the most pronounced excursions from 0, that is, the most signal relative to noise.

To account for an incomplete counterbalancing of stimulation locations over sessions, we repeated all ANOVAs to include “session order” as a covariate, with the exception of the ERP data. Whenever there was a significant interaction of a factor with this covariate, we report statistics adjusted for including this covariate.

## 3 Results

### 3.1 tVNS Is Not Different in Perceived Intensity, Pleasurableness Across Stimulation Locations

**FIGURE 2:**
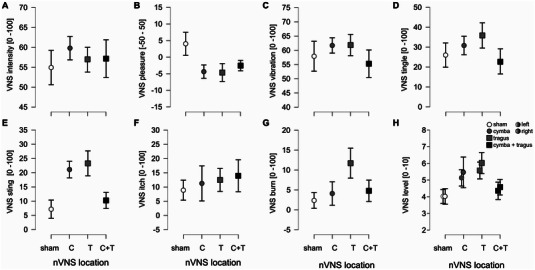
Participants’ sensations of transcutaneous vagus nerve stimulation (tVNS) stimulation on the ear. Graphs show average ratings ± standard error of the mean (SEM) across participants with respect to each tVNS stimulation location (on the *x*‐axis). The tVNS stimulation locations are indicated with different symbols, open circles—sham/ear lobe, black circles—cymba conchae, open square—tragus, black square—cymba conchae and tragus. The different sensations (panels A: intensity, B: pleasure, C: vibration, D: tingle, E: sting, F: itch, G: burn) that were rated on Visual Analog Scales (VAS) are in separate graphs, labeled on the *y*‐axis. Panel H shows the vagus nerve stimulation (VNS) level in mA for the left and right ear separately. Significant planned follow‐up *t*‐tests are indicated with an asterisk (*p* < 0.05, Holm corrected for multiple comparisons).

After placement of the electrodes, the intensity (amplitude) of stimulation was adjusted to the perceptual target of “as intense as possible without getting uncomfortable” (see Section 2) and the amplitude setting recorded. The adjusted amplitude was not different between stimulation locations (*F*[3,24] = 2.066, *p* = 0.131) and also not different for right ear and left ear (see Supporting Information Tables 1 and 2). Participants then also rated the sensations of tVNS stimulation on the ear with VAS. The average intensity, pleasantness, burning, stinging, itching, tingling, and vibration sensations are displayed in Figure 2 and Supporting Information Table 3. As intended, the perceived intensity did not differ between tVNS conditions (Supporting Information Tables 4–6), however, stronger stinging sensations were observed for the tragus (*p* = 0.028) relative to sham. We note that the average stinging sensations remained below ∼20% on the VAS. Other sensations associated with tVNS on the ear did not differ as a function of stimulation location (see Supporting Information Tables 4–6). The average time since the last meal was 2.34 h (± 0.89) and did not differ by location (*F*[3,39] = 0.131, *p* = 0.849).

Participants also rated their internal and other state sensations on VAS (see Figure 2) and none of these varied as a function of stimulation location at the start of the session (see Figure 4). These results show that tVNS stimulation location and contextual factors generally did not cause participants to feel any differently at the start of the experiment.

### 3.2 tVNS Location Does Not Affect Spontaneous Eye‐Blink Rate, but Sipping a Milkshake Does

**FIGURE 3:**
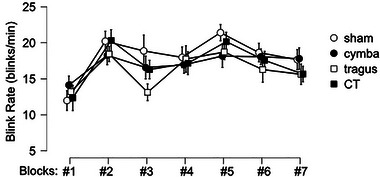
Participants’ spontaneous eye‐blink rate as a function of transcutaneous vagus nerve stimulation (tVNS) stimulation on the ear. Graphs show the average number of eye blinks per minute ± standard error of the mean (SEM) across participants per tVNS stimulation location. The tVNS stimulation locations are indicated with different symbols, open circles—sham/ear lobe, black circles—cymba conchae, open square—tragus, black square—cymba conchae and tragus. The *x*‐axis reflects the factor block (Blocks #1–7). Significant planned follow‐up *t*‐tests are indicated with an asterisk (*p* < 0.05, Holm corrected for multiple comparisons).

To test whether dopamine tone as reflected by eye‐blink rate differed as a result of timing of tVNS or tVNS location, we analyzed the average eye‐blink count per minute in a 4 × 7 repeated‐measures ANOVA with the factors stimulation location (L, C, T, CT) and block (from #1 to #7). The average blink rate per minute for the different tVNS conditions during each block are displayed in Figure 3 and Supporting Information Table 7. During the EEG recordings, we did not observe an effect of tVNS stimulation location on eye‐blink rate (*F*[3,42] = 0.927, *p* = 0.436, *η*_p_^2^ = 0.062, Supporting Information Table 8). We did observe an effect of block on eye‐blink rate (*F*[6,84] = 6.641, *p* < 0.001, *p*_FDR_ = 0.002, *η*_p_^2^ = 0.322). As can be seen in Figure 3, eye‐blink rate increased mostly during the first sipping block (which was before tVNS stimulation was turned on). Inspection of post hoc planned comparisons (Supporting Information Table 9) showed that this effect was driven by increases in eye‐blink rate after the first resting block; Block #1 is significantly different from all the other blocks (*p* < 0.05 for Block #7, *p* < 0.01 for Blocks #4 and #6, *p* < 0.001 for #2 and #5) except for Block #3 (*p* = 0.133). There was no interaction between tVNS stimulation location and block on eye‐blink rate (*F*[18,252] = 1.135, *p* = 0.318, *η*_p_^2^ = 0.075). These results show that tVNS stimulation location did not increase eye‐blink rate, but that sipping of a milkshake caused a transient shift in SEBR that lasted for the duration of the session.

### 3.3 Internal State Changes Over the Course of the Session, but Is Not Different Between Stimulation Locations

**FIGURE 4:**
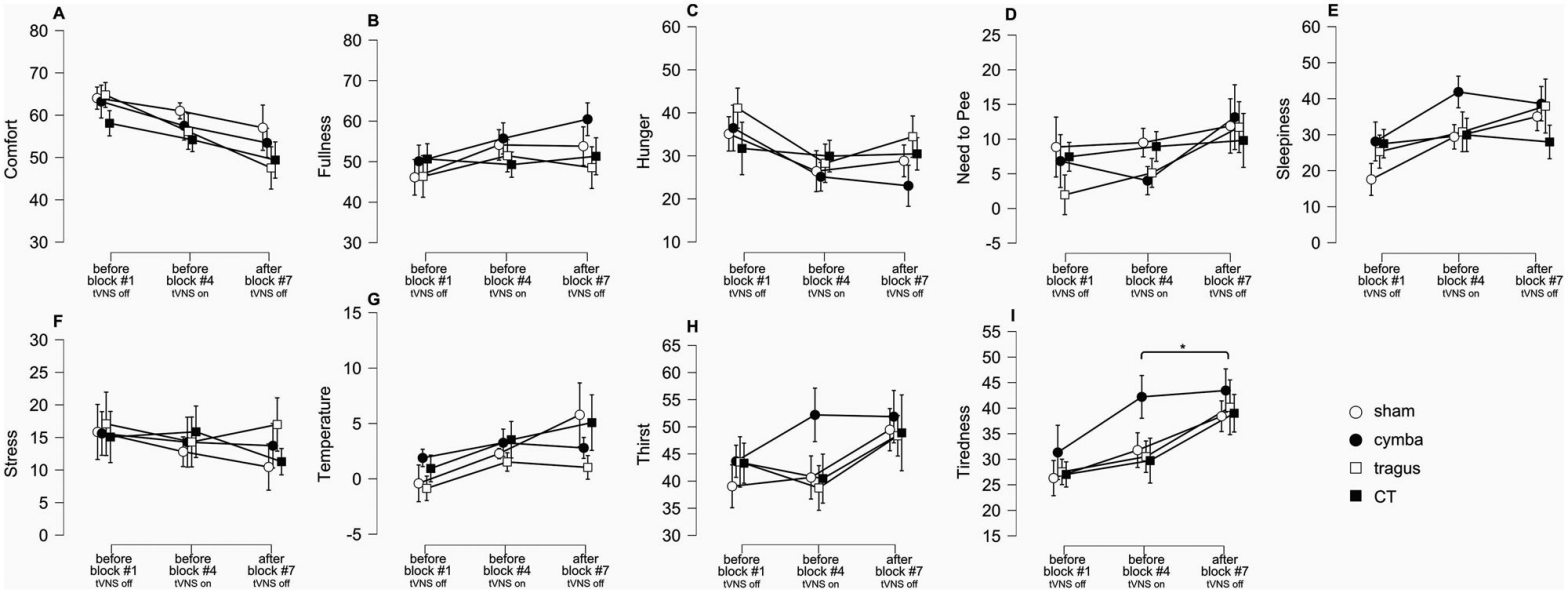
Participants' sensations of internal and other states. Graphs show average ratings ± standard error of the mean (SEM) across participants per transcutaneous vagus nerve stimulation (tVNS) stimulation location. The tVNS stimulation locations are indicated with different symbols and lines, open circles—sham/ear lobe, black circles—cymba conchae, open square—tragus, black square—cymba conchae and tragus. The *x*‐axis reflects the factor time (“Before Block #1, tNVS off,” “Before Block #4, tVNS on,” “After Block #7, tVNS off”). The different sensations (panels A: comfort, B: fullness, C: hunger, D: need to pee, E: sleepiness, F: stress, G: temperature, H: thirst, I: tiredness) that were rated on VAS are in separate graphs, labeled on the *y*‐axis. Significant planned follow‐up *t*‐tests are indicated with an asterisk (*p* < 0.05, Holm corrected for multiple comparisons).

Before the EEG recording started, after the participant was comfortably seated in the recording room, we asked them to report their internal states and perception of environmental conditions (“Before Block #1, tNVS off”). The participant was asked to report these again after completing Blocks 1–3 and the VNS stimulation was started (“Before Block #4, tVNS on”). A third set of ratings was obtained after the EEG recording stopped, at the end of the session (“After Block #7, tVNS off”). The average internal state ratings are displayed in Figure 4 (see Supporting Information Tables 10–12). We did not observe an effect of tVNS stimulation location on any of these ratings. We observed an effect of time on tiredness (*F*(2,26) = 10.202, *p* < 0.001, *p*_FDR_ = 0.015, *η*_p_^2^ = 0.44). Post hoc planned comparison showed that comfort tiredness increased from “Before Block #4, tVNS on” to “After Block #7, tVNS off” (post hoc *p* = 0.04). The interaction between time and location was not significant for any of the internal state and environment ratings. These analyses show that during all sessions, participants felt approximately similar about their internal state and their environment, including how the testing session affected them.

### 3.4 Perception of the Chocolate Milk Does Not Change With Stimulation

**FIGURE 5:**
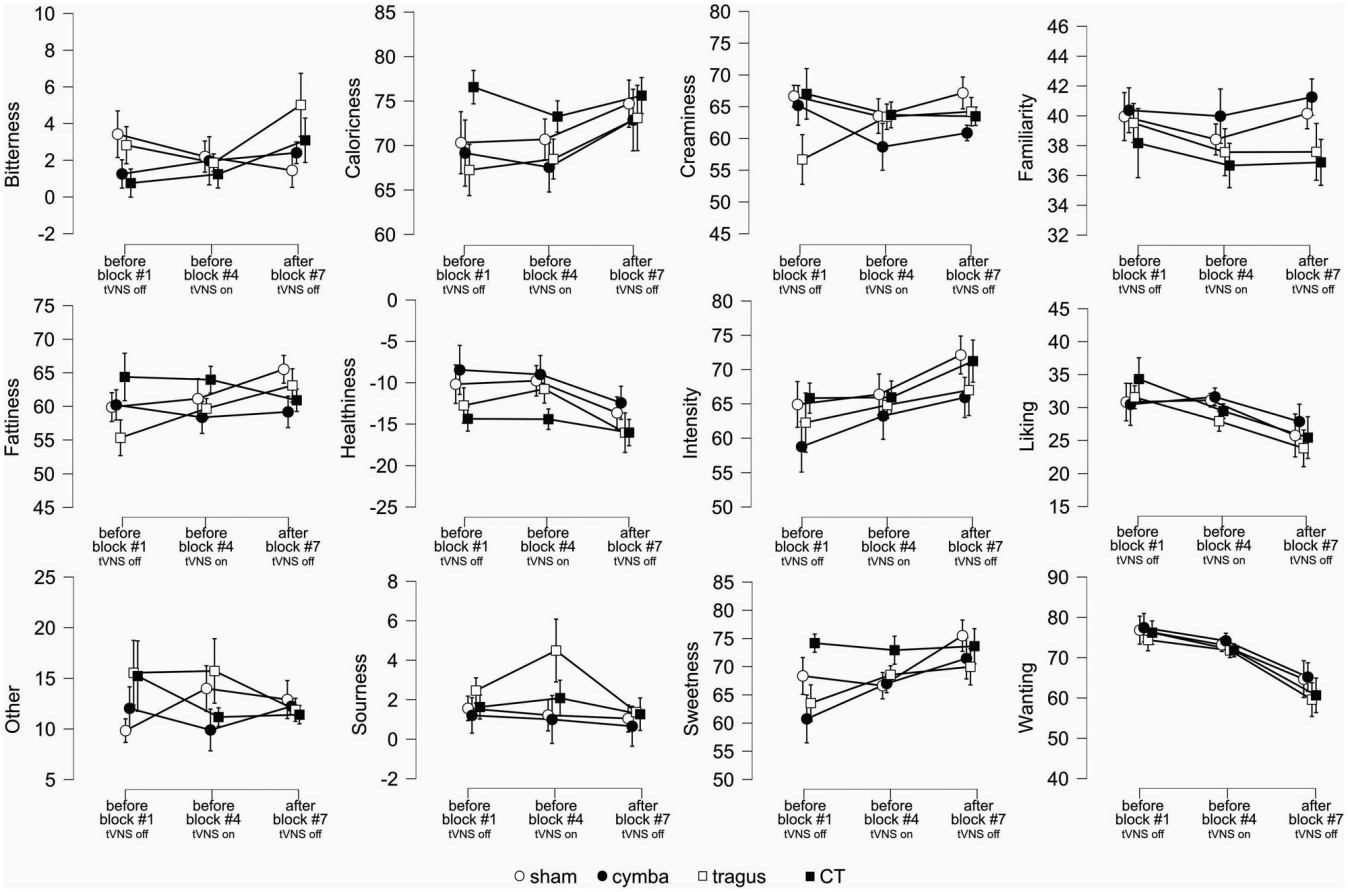
Participants’ perception of the chocolate milk as a function of transcutaneous vagus nerve stimulation (tVNS) stimulation on the ear. Graphs show average ratings ± standard error of the mean (SEM) across participants per tVNS stimulation location. The tVNS stimulation locations are indicated with different symbols, open circles—sham/ear lobe, black circles—cymba conchae, open square—tragus, black square—cymba conchae and tragus. The *x*‐axis reflects the factor time (“Before Block #1, tNVS off,” “Before Block #4, tVNS on,” “After Block #7, tVNS off”). The different sensations (panels A: Bitterness, B: Caloricness, C: Creaminess, D: Familiarity, E: Fattiness, F: Healthiness, G: Intensity, H: Liking, I: Other, J: Sourness, K: Sweetness, L: Wanting) that were rated on VAS are in separate graphs, labeled on the *y*‐axis. Significant planned follow‐up *t*‐tests are indicated with an asterisk (*p* < 0.05, Holm corrected for multiple comparisons).

Participants were asked to rate the chocolate milk before the start of EEG recording (“before Block #1, tNVS off”), during the recording (“before Block #4, tVNS on”), and at the end of recording (“after Block #7, tVNS off”). Each time they were given a single sip and then asked to rate the chocolate milk on 12 different scales (Bitterness, Caloricness, Creaminess, Familiarity, Fattiness, Healthiness, Intensity, Liking, Sourness, Sweetness, Wanting, and Other). The average drink perception ratings for the different tVNS conditions during each time point are displayed in Figure 5 and Supporting Information Table 13. After correction for multiple tests, we did not observe an effect of tVNS stimulation location on any of these ratings (see Supporting Information Table 14). We did not observe an effect of time for any of the ratings, nor for the interaction of time and location (see Supporting Information Tables 14–16). In summary, generally there are no effects of time or stimulation location on the perception of the drink.

### 3.5 Sip Weight Is Not Affected by Stimulation

**FIGURE 6:**
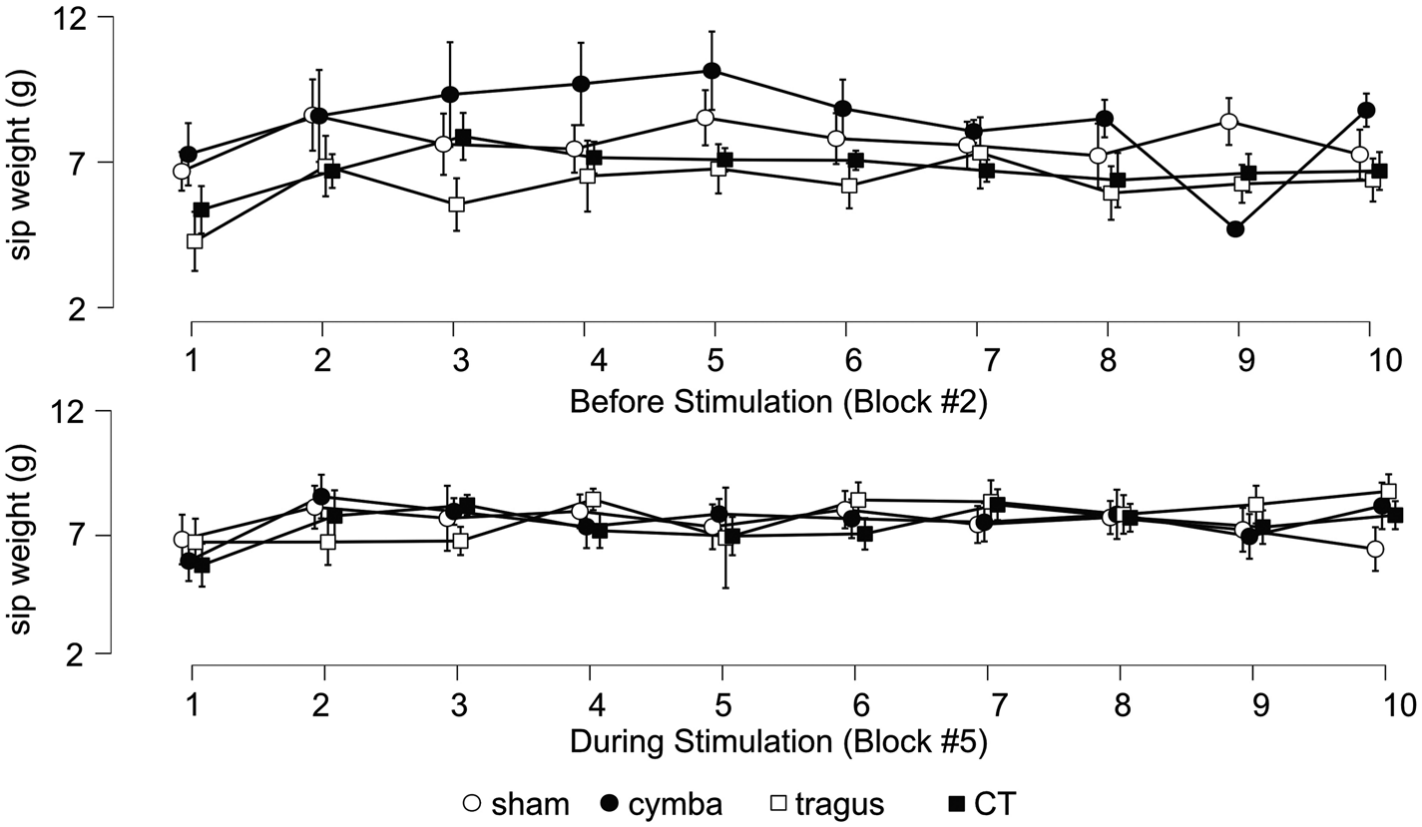
Participants’ consumed amount of the chocolate milk during the sipping blocks as a function of transcutaneous vagus nerve stimulation (tVNS) stimulation on the ear. Graphs show average sip weight in g ± standard error of the mean (SEM) across participants per tVNS stimulation location. The tVNS stimulation locations are indicated with different symbols, open circles—sham/ear lobe, black circles—cymba conchae, open square—tragus, black square—cymba conchae and tragus. The *x*‐axis reflects the factor sipping cycle (1–10). The different panels represent the two sipping blocks, A: Block #2, sipping, tVNS off and B: Block #5, sipping, tVNS on. Significant planned follow‐up *t*‐tests are indicated with an asterisk (*p* < 0.05, Holm corrected for multiple comparisons).

To ensure that participants were sipping the same amount of the chocolate milk during the sipping blocks, we extracted the change in weight between before and after each sipping cycle within each block. The average scale changes for the different tVNS conditions during each sipping block and each sipping cycle within a block are displayed in Figure 6 and Supporting Information Table 18. We then specified a repeated‐measures ANOVA with factors block (Block #2, sipping, tVNS off,'' “Block #5, sipping, tVNS on”), location (L, C, T, CT), and cycle (1–10). Two participants had missing data so a total of 13 participants were included in this analysis. The effect of block was not significant (*F*(1,1.82) = 0.202, *p* = 0.661, *η*_p_^2^ = 0.055), nor was the effect of location (*F*(1.82,21.835) = 0.696, *p* = 0.496, *η*_p_^2^ = 0.013), nor the effect of cycle (*F*(2.217,26.606) = 2.362, *p* = 0.109, *η*_p_^2^ = 0.164). The interaction between block and location was not significant (*F*(1.886,22.627) = 2.631, *p* = 0.097, *η*_p_^2^ = 0.180) and the trend in this interaction seems mostly driven by differences between the different locations in Block #2, before the VNS stimulator was turned on. The interaction between block and cycle was not significant (*F*(2.409,28.913) = 0.924, *p* = 0.424, *η*_p_^2^ = 0.071), nor was the interaction between location and cycle (*F*(27,324) = 1.117, *p* = 0.318, *η*_p_^2^ = 0.085), nor the three‐way interaction (*F*(27,324) = 0.579, *p* = 0.956, *η*_p_^2^ = 0.046). These analyses show that chocolate milk intake generally was not different as a result of timing of tVNS, nor as a result of different tVNS locations.

### 3.6 Swallowing Behavior Does Not Change With Stimulation Location

**FIGURE 7:**
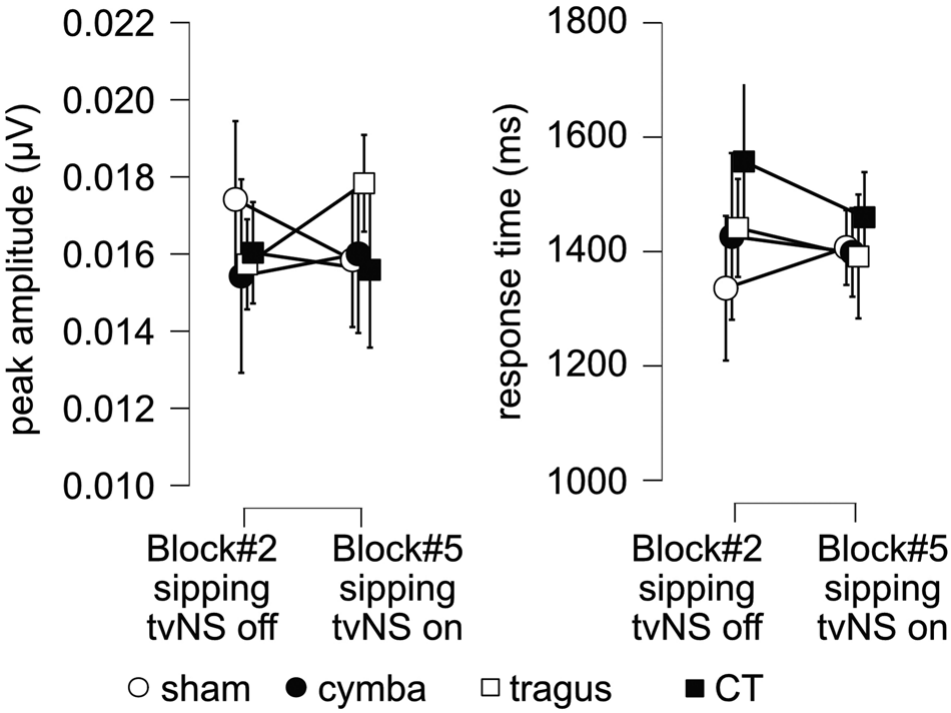
Participants’ swallowing behavior during the sipping blocks as a function of transcutaneous vagus nerve stimulation (tVNS) stimulation on the ear. Graphs show average parameters ± standard error of the mean (SEM) across participants per tVNS stimulation location. The tVNS stimulation locations are indicated with different symbols, open circles—sham/ear lobe, black circles—cymba conchae, open square—tragus, black square—cymba conchae and tragus. The *x*‐axis reflects the factor block: “Block #2, sipping, tVNS off” and “Block #5, sipping, tVNS on.” The different panels represent the two parameters we extracted, A: peak amplitude of the electromyogram (EMG) signal and B: latency of the peak amplitude.

To ensure that participants were swallowing in a similar manner in the sipping blocks, we analyzed extracted swallowing components from the submental EMG signal and averaged over the sips within each block. The average swallowing measures for the different tVNS conditions during each sipping block are displayed in Figure 7 and Supporting Information Tables 17 and 18. We ran a repeated‐measures ANOVA with within‐subjects factors block (“Block #2, sipping, tVNS off,” “Block #5, sipping, tVNS on”) and location (sham, C, T, CT) on the following swallowing components: (1) peak latency relative to cue, and (2) peak amplitude. For the analysis with peak latency relative to cue we observed no main effect of block (*F*(1,14) = 0.219, *p* = 0.647, *η*_p_^2^ = 0.015), no main effect of stimulation location (*F*(3,42) = 0.335, *p* = 0.8, *η*_p_^2^ = 0.023) and no interaction for block and stimulation location (*F*(3,42) = 0.571, *p* = 0.637, *η*_p_^2^ = 0.039). For the analysis of peak amplitude, we observed no main effect of block (*F*(1,14) = 0.02, *p* = 0.891, *η*_p_^2^ = 0.001), no main effect of stimulation location (*F*(3,42) = 0.129, *p* = 0.942, *η*_p_^2^ = 0.009) and no interaction effect of block and stimulation location (*F*(3,42) = 0.589, *p* = 0.626, *η*_p_^2^ = 0.040). These analyses confirm that swallowing behavior generally was not different as a result of stimulation, nor as a result of different stimulation locations (Figure 7).

### 3.7 ERP and Spatiotemporal Analyses Show No Difference Between tVNS Location on Neural Responses to a Chocolate Milk During Sipping Blocks

From a total of 5837 trials successfully registered with a swallow signal, 4969 (85.1%) remained after preprocessing and cleaning the data. We specified a 2 × 4 within‐subjects ANOVA with within‐subjects factors block (“Block #2, sipping, tVNS off,” “Block #5, sipping, tVNS on”) and tVNS stimulation location (sham, C, T, CT). We observed no main effect of block (44 clusters detected, all *p*’s > 0.209), no main effect of location (two clusters detected, *p*’s > 0.415) and no interaction between block and location (47 clusters detected, corrected *p*’s > 0.583). We then performed paired *t*‐tests between all tVNS stimulation locations in “Block #5, sipping, tVNS on.” We observed one trend toward a significant difference in tragus relative to sham in electrode F8 at ∼200 ms poststimulus (corrected *p* = 0.0672, Supporting Information Table 21). In all other pairwise comparisons between conditions, we observed no differences (all *p*’s > 0.2044, Supporting Information Table 21). We then examined *t*‐tests against zero for each tVNS stimulation location and each sipping block. During “Block #2, sipping, tVNS off” in all stimulation locations, we observed significant excursions from 0 around 450–550 ms postswallow in various electrodes, usually in midline or right hemisphere frontal, occipital, and parietal electrodes (Supporting Information Table 20). During “Block #5, sipping, tVNS on” we generally observed more significant excursions from zero, particularly in the tragus stimulation location, where we see responses in both left and right hemisphere and midline frontal, occipital, and parietal electrodes at 450–550 ms postswallow. These analyses indicate that there are no effects of stimulation location on flavor‐evoked ERPs.

## 4 Discussion

The goal of this study was to screen for the optimal location(s) for tVNS in the context of oral food rewards. We used various psychological and neurophysiological measures before, during, and after sipping a chocolate milk as well as before and during tVNS stimulation in four different stimulation locations (sham in earlobe, cymba conchae, tragus, and cymba conchae and tragus). We generally observed no effects of the ad verum stimulation conditions (cymba conchae, tragus, and cymba conchae and tragus) relative to sham, for example, for the SEBR and for the sipping ERPs. Our control variables of internal and environmental state, tVNS sensation, sip size, and swallowing behavior did not show main effects of tVNS stimulation location either. We observed no effects of stimulation location on the perception of the chocolate milk or on ERP's during sipping of the milkshake. Unexpectedly, we observed that sipping the chocolate milk increased SEBR.

We observed no effect of tVNS location on the ERPs. One of the possible reasons for the lack of significant differences between conditions can be the sample size. While we observed a trend for a difference in tragus versus sham around 200 ms postswallow in a frontal electrode, the statistical analyses pointed out that the differences are not enough and that this may be a false positive. The ERP analyses were time‐locked to the peak in the swallow EMG signal. It is not clear how long after the peak in EMG swallow signal the retronasal olfactory pathway is stimulated and this may be later than 200 ms postswallow peak. It is also possible that fat and sweet signals may contribute to this, because oral receptors are already stimulated before swallowing. This trend for a greater negativity at 200 ms for tragus stimulation may or may not prove to be significant with a larger sample size. We recommend that future studies may utilize a paradigm that is known to evoke robust N2 responses, for example, with an oddball paradigm (Patel and Azzam 2005) or with a Go–Nogo task that was previously shown to decrease N200 with tVNS (Pihlaja et al. 2020).

Another possibility for observing no effects of tVNS stimulation is that EEG is not sensitive enough to measure tVNS modulation of food reward areas, as these areas are typically deeper in the brain. fMRI studies on the effect of tVNS on food processing indeed confirm involvement of predominantly deeper brain areas, such as amygdala, insula, operculum, caudate, ventral striatum, pre‐ and postcentral gyrus, cingulate gyrus (see Table 1 in Teckentrup et al. 2021). Another study that looked at the effect of tVNS on EEG with food stimuli (images; Obst et al. 2020) observed only general tVNS effects. They did not observe any interaction between image type (food vs. object) and tVNS stimulation, which may also be because of interactions in deeper lying brain areas.

It is also possible that there are more robust effects on oscillations of the EEG signal, but a power spectrum analysis is beyond the scope of the current manuscript and will be reported elsewhere.

We observed no changes in the perception of the chocolate milk as a result of stimulation location, unlike our previous studies (Öztürk et al. 2020; Altınkaya et al. 2023). This may be the result of methodological differences, including the absence of a rating task during sipping in the current report. Perhaps the conscious evaluation of an oral stimulus enhances effects of tVNS and future studies may directly investigate this. As noted by others (Koepp et al. 2021), tVNS may have a normalization effect, and thus increases may be observed under low liking or wanting conditions, decreases under high liking or wanting, and no effects in between. This may be an explanation for our lack of effects of tVNS on liking or wanting here, as liking of the stimulus was around ∼ 30 points on a scale ranging from −50 to +50.

We observed an increase in SEBR in the first sipping block, which persists throughout the entire session. One of the roles of dopamine in food reward is that phasic stimulus‐driven release increases reflect a positive reward prediction error (Schultz, Dayan, and Montague 1997; Maia and Frank 2011). SEBR correlates positively with reward prediction errors in eating disorders (Frank et al. 2020) and the amount of willingness to work (Pas et al. 2014). This means that participants may have experienced a positive prediction error and increased motivation as a result of increases in phasic dopamine in the brain. To our awareness, we are the first to report an increase in SEBR as a result of food consumption in humans. Most studies in humans measuring SEBR use cognitive manipulation (Colzato and Beste 2020), and doubt has been shed on the usefulness of SEBR as a reflection of phasic dopamine shifts (Dang et al. 2017). However, it is also possible that the usual cognitive manipulations are too subtle or do not evoke positive enough reward. Future studies should systematically study hunger versus full to get a better assessment of SEBR responses to food. The potentially small effect of tVNS stimulation location on SEBR may be completely overshadowed by the effect that food consumption has in our design. To more fully assess tVNS effects on SEBR, a study where tVNS precedes food consumption is critical.

There are a few limitations to this study. We had a relatively small sample size, which was powered to detect large effect sizes like we observed in one of our previous studies (Öztürk et al. 2020), but not medium or small effects. We do note that power analyses from small samples (11 participants in the study by Öztürk et al.) tend to underestimate the needed sample size (Albers and Lakens 2018). Despite a small sample size, we were still able to detect robust effects of, for example, an increase in SEBR due to chocolate milk consumption. It is possible that we missed more subtle effects from stimulation location due to this small sample size. This means that the null effects that we observe as a result of tVNS must be confirmed in a larger sample, but also that any tVNS effects are going to be smaller and potentially less meaningful than effects of, for example, food consumption.

How can we be sure that we are activating the ABVN? There is no established manipulation check variable for tVNS studies or a biomarker for ABVN activation. Despite the suggestion of HRV as a potential biomarker (among others, such as salivary alpha amylase, somatosensory‐evoked potential, P300, and pupil dilation), it is not accepted as valid measure of VN stimulation (Borges and Laborde 2024; Burger et al. 2020), and Burger et al. (2020) state: “Although changes in HRV may be a sufficient requirement for demonstrating vagal activation, it is unlikely to be a necessary requirement for the activation of afferent vagal fibers.” Underlining this is the living meta‐analysis of tVNS effect on HRV measures (Wolf et al. 2021), which shows that when we select studies with healthy participants comparing tVNS to sham stimulation in within‐participant designs, there is heterogeneity among studies, with some showing increases in HRV, others showing decreases, and many showing no effects. The meta‐analytic effect of tVNS on RMSSD (Root Mean Square of Succesive Differences, the most commonly used measure of HRV) is Hedge's *g* = −0.008 (95% credible interval [−0.136 to 0.123]). Finding a reliable biomarker is an important goal in the field (Borges and Laborde 2024), and it is important to recognize the lack of a biomarker in these studies. However, even if HRV were a reliably biomarker, it may not have been insightful in our study, since fasting increases HRV, and food consumption decreases HRV (Rominger et al. 2021), and our food stimulation condition that precedes tVNS may have overshadowed any effects of tVNS on HRV. In line with this, in our previous study we observed tVNS‐induced decreases in HRV prefood consumption, but equal to sham postfood consumption. In our current study, we have no way of directly assessing if the ABVN was stimulated. This is the case for the majority of tVNS studies. However, we do know that we are stimulating the nerves in the ear, due to the reported sensations by participants. For example, the intensity of tVNS sensation across the different stimulation locations was between ∼ 55 and 60 on a 101‐point VAS, with coefficients of variation between 0.22 and 0.28, see also Figure 2. We stimulate in the areas associated with ABVN innervation (C, T, CT) or greater auricular nerve innervation (lobe and tail of helix; Peuker and Filler 2002). We also know that tVNS generally stimulates VN brainstem targets including the NST as evaluated by fMRI responses in a similar way that invasive VNS does (Frangos et al. 2015). In addition, there is meta‐analytic evidence of tVNS perturbing downstream targets of the NST (Rajiah et al. 2024). Therefore, we may infer, but cannot directly prove that stimulation of the afferent fibers of the ABVN was achieved. However, as the afferent outputs from the gut‐to‐the‐brain are a much larger portion of the neurons in the VN than the fibers of the ABVN, it may be expected that any effective ABVN stimulation will only ever be capable of exerting small influences. Therefore, studies with a larger sample size may be useful.

Another important limitation is that we did not turn on tVNS stimulation until after the first sipping blocks, which may have induced ceiling effects in some dependent variables that may have made it impossible to observe subsequent tVNS effects. In a parallel study that compared the same tVNS stimulation locations, we observed most effects before food consumption, for example, a decrease in wanting and a decrease in HRV (Altınkaya et al. 2023). This may be related to the relatively small portion of VN fibers that come from the ABVN compared to those from the gut that are already carrying important information to the brain regarding the caloric and macronutrient composition of the chocolate milk. We recommend that future studies start delivering tVNS before food stimulation.

In conclusion, we observe no clear optimal ear location for tVNS‐induced modulation of ERP from EEG, eye‐blink rate, perceptual and hedonic aspects of flavor, swallowing behavior, and consumption behavior in this study. We did observe an effect of food consumption on eye‐blink rate, which future studies may confirm to be a sensitive proxy for food reward‐related phasic dopamine shifts.

## Author Contributions

**Samet Albayrak**: data curation, formal analysis, investigation, methodology, visualization, software, writing – review and editing, writing – original draft, project administration. **Berfin Aydin**: investigation, methodology, writing – review and editing. **Gizem Özen**: writing – review and editing, investigation. **Faruk Yalçin**: writing – review and editing, investigation. **Merve Balık**: investigation, writing – review and editing. **Hüseyin Yanık**: methodology, software, writing – review and editing. **Burcu A. Urgen**: conceptualization, writing – review and editing, resources. **Maria Geraldine Veldhuizen**: conceptualization, supervision, funding acquisition, writing – review and editing, software, resources.

## Ethics Statement

The Mersin University Committee for Clinical Research approved the study protocol (number 7807789/050.01.04/1152384) and written informed consent was obtained from all study participants.

## Conflicts of Interest

The authors declare no conflicts of interest.

### Peer Review

The peer review history for this article is available at https://publons.com/publon/10.1002/brb3.70355.

## Supporting information



Supporting Information

## Data Availability

The data of this study will be made publicly available in the Open Science Framework at https://osf.io/uvax7/.
