# Supplementary material for: Transcutaneous Vagus Nerve Stimulation Effects on Flavor‐Evoked Electroencephalogram and Eye‐Blink Rate
Source: Brain Behav. 2025 Mar 13;15(3):e70355. doi: 10.1002/brb3.70355 (PMC11904970; doi:10.1002/brb3.70355)
Supplement: Supplementary file 1 — Supporting Information [file BRB3-15-e70355-s001.docx]

**Supplementary Materials**

**Supplementary Figure 1**

| 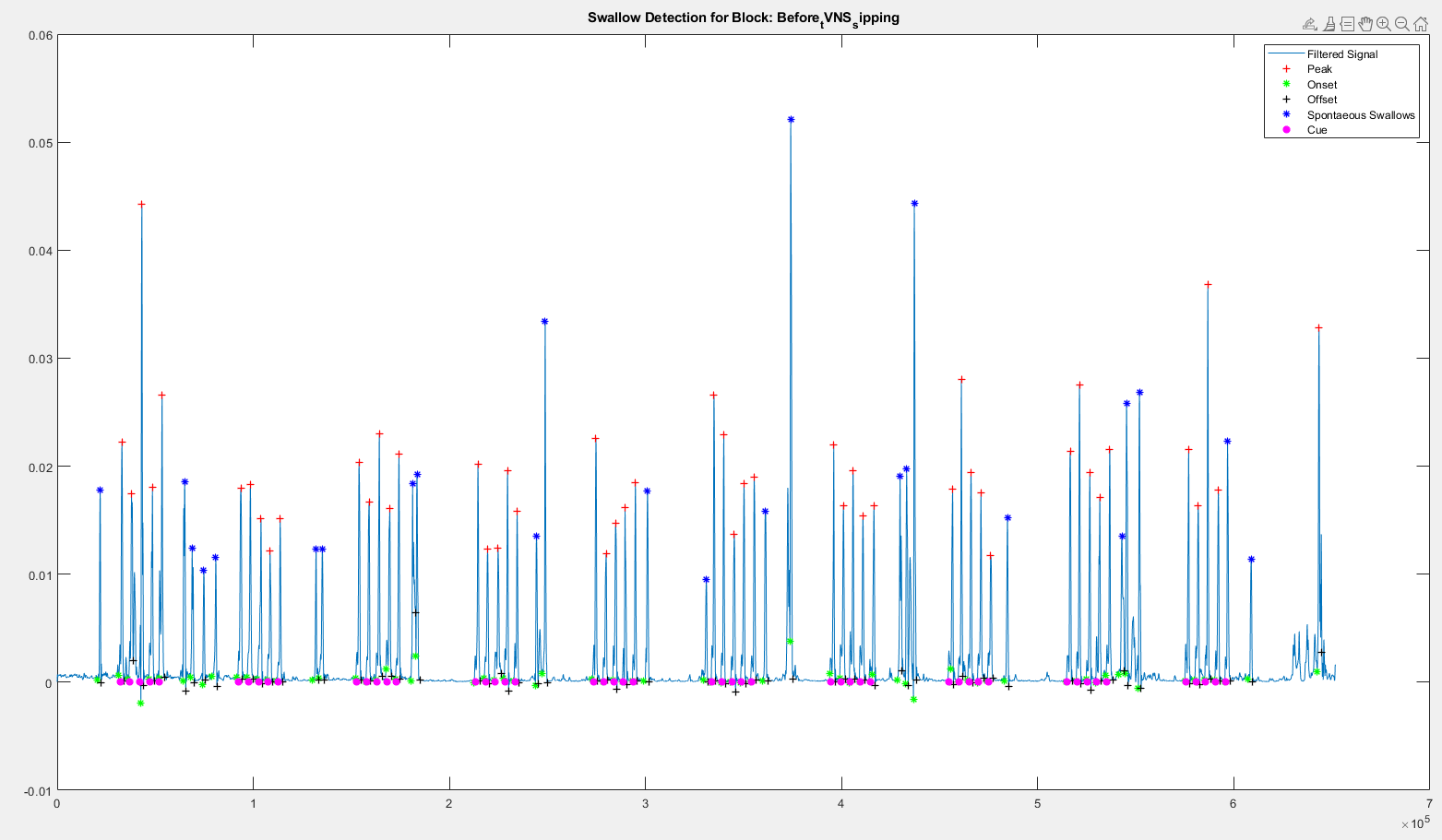 |
| --- |
| Example of extraction of swallows for one sipping block. In blue we plotted the EMG signal in μV (y-axis) against time (in ms, x-axis). Pink circles indicate the sip-cues, red plusses indicate the swallow peak post-cue, while blue stars indicate spontaneous swallows that were not preceded by a sip-cue. From the peaks the onsets (green circle) and offset (black plus) of each swallow were identified. and in ne block of swallow markers extracted form EMG data shown on amplitude vs time scale with each auditory cue, swallow moment and spontaneous swallows marked. From the position of these markers we calculated peak-latency post-cue, peak amplitude and area under the curve between onset and offset. |

**Supplementary Table 1**

Descriptive statistics of nVNS stimulation in mA per location

| **VNS Level** | **Sham mean (+/-sd)** | **C mean (+/-sd)** | **T mean (+/-sd)** | **CT mean (+/-sd)** |
| --- | --- | --- | --- | --- |
| **Left Ear** | 4 (2.18) | 5.11 (2.76) | 5.56 (2.65) | 4.33 (2.78) |
| **Right Ear** | 4 (2.12) | 5.44 (4.33) | 6 (2.35) | 4.56 (2.88) |

**Supplementary Table 2**

Statistics of ANOVA of effect of location on stimulation level on left or right ear

| **VNS Level** | **F** | **df** | **P-value** | **η_p_^2^** |
| --- | --- | --- | --- | --- |
| **Location** | 2.066 | (3,24) | 0.131 | 0.205 |
| **L-R** | 3.273 | (1,8) | 0.108 | 0.29 |
| **Loc * L-R** | 0.143 | (3,24) | 0.933 | 0.018 |

**Supplementary Table 3**

Planned comparison t-tests for effect of location on intensity (amplitude in mA) of stimulation selected by the participant

|  | **vs Sham** | **vs Cymba** | **vs Tragus** |
| --- | --- | --- | --- |
| **Sham** | . | . | . |
| **Cymba** | t =-1.62, p = ns, [-3.55 - 0.99] | . | . |
| **Tragus** | t =-2.254, p = ns, [-4.05 - 0.49] | t =-0.634, p = ns, [-2.77 - 1.77] | . |
| **CT** | t =-0.563, p = ns, [-2.71 - 1.82] | t =1.056, p = ns, [-1.44 - 3.1] | t =1.69, p = ns, [-0.94 - 3.6] |

*Holm corrected for 6 comparisons

**Supplementary Table 4**

Descriptive statistics of perceptual ratings of nVNS stimulation per location

| **VAS label** | **Sham** | **C** | **T** | **CT** |
| --- | --- | --- | --- | --- |
|  | **mean (+/-sd)** | **mean (+/-sd)** | **mean (+/-sd)** | **mean (+/-sd)** |
| Intensity | 59.62 (13.38) | 59.07 (14.32) | 58.91 (16.38) | 56.47 (15.75) |
| Pleasantness | -1.88 (20.55) | -4.84 (19.61) | -4.12 (22.42) | -6.66 (22.07) |
| Sting | 4.65 (8.75) | 17.01 (21.03) | 20.05 (21.22) | 10.9 (21.15) |
| Burn | 3.38 (5.67) | 5.91 (11.67) | 8.19 (18.97) | 6.48 (17.33) |
| Tingle | 37.71 (28.33) | 37.41 (25.23) | 43.05 (27.64) | 37.25 (24.64) |
| Itching | 8.57 (20.58) | 16.38 (21.24) | 12.72 (23.56) | 9.38 (17.71) |
| Vibration | 66.16 (19.72) | 59.11 (22.95) | 67.75 (16.08) | 63.99 (21.15) |

**Supplementary Table 5**

Statistics of ANOVA of effect of location on perceptual ratings of nVNS stimulation

| **VAS label** | **F (3,36)** | **P-value** |
| --- | --- | --- |
| Intensity | 0.163 | 0.921 |
| Pleasantness | 0.967 | 0.417 |
| Sting | 3.524 | 0.023 (p_FDR_ = 0.161)* |
| Burn | 0.466 | 0.707 |
| Tingle | 0.263 | 0.852 |
| Itching | 0.187 | 0.187 |
| Vibration | 1.867 | 0.15 |

* corrected for false discovery rate with the Benjamini-Hochberg procedure for a family of 7 tests

**Supplementary Table 6**

Statistics of planned comparison p-values for t-tests of perceptual ratings of VNS per location

| **VAS label** | **Location** | **t-value, p-value*, [95% CI]** | | |
| --- | --- | --- | --- | --- |
|  |  | **vs sham** | **vs C** | **vs T** |
| **Intensity** | Sham |  |  |  |
|  | C | t =0.112, p = ns, [-13.04 - 14.15] | . | . |
|  | T | t =0.145, p = ns, [-12.88 - 14.31] | t =0.032, p = ns, [-13.44 - 13.75] | . |
|  | CT | t =0.643, p = ns, [-10.44 - 16.75] | t =0.531, p = ns, [-10.99 - 16.2] | t =0.498, p = ns, [-11.15 - 16.04] |
| **Pleasantness** | Sham |  |  |  |
|  | C | t =1.039, p = ns, [-4.92 - 10.83] | . | . |
|  | T | t =0.787, p = ns, [-5.64 - 10.11] | t =-0.252, p = ns, [-8.59 - 7.16] | . |
|  | CT | t =1.682, p = ns, [-3.09 - 12.66] | t =0.642, p = ns, [-6.05 - 9.7] | t =0.894, p = ns, [-5.33 - 10.42] |
| **Sting** | Sham |  |  |  |
|  | C | t =-2.403, p = ns, [-26.6 - 1.88] | . | . |
|  | T | **t =-2.994, p = 0.028, [-29.64 - -1.16]** | t =-0.591, p = ns, [-17.28 - 11.2] | . |
|  | CT | t =-1.215, p = ns, [-20.49 - 7.99] | t =1.188, p = ns, [-8.13 - 20.35] | t =1.779, p = ns, [-5.09 - 23.39] |
| **Burn** | Sham |  |  |  |
|  | C | t =-0.612, p = ns, [-13.95 - 8.9] | . | . |
|  | T | t =-1.166, p = ns, [-16.24 - 6.62] | t =-0.554, p = ns, [-13.72 - 9.14] | . |
|  | CT | t =-0.751, p = ns, [-14.53 - 8.33] | t =-0.139, p = ns, [-12 - 10.85] | t =0.415, p = ns, [-9.72 - 13.14] |
| **Tingle** | Sham |  |  |  |
|  | C | t =0.039, p = ns, [-21.11 - 21.72] | . | . |
|  | T | t =-0.69, p = ns, [-26.76 - 16.08] | t =-0.73, p = ns, [-27.06 - 15.77] | . |
|  | CT | t =0.06, p = ns, [-20.95 - 21.88] | t =0.021, p = ns, [-21.25 - 21.58] | t =0.751, p = ns, [-15.61 - 27.22] |
| **Itching** | Sham |  |  |  |
|  | C | t =-1.948, p = ns, [-18.91 - 3.29] | . | . |
|  | T | t =-1.034, p = ns, [-15.25 - 6.96] | t =0.914, p = ns, [-7.44 - 14.77] | . |
|  | CT | t =-0.201, p = ns, [-11.91 - 10.3] | t =1.747, p = ns, [-4.1 - 18.11] | t =0.834, p = ns, [-7.76 - 14.44] |
| **Vibration** | Sham |  |  |  |
|  | C | t =1.812, p = ns, [-3.72 - 17.83] | . | . |
|  | T | t =-0.409, p = ns, [-12.37 - 9.18] | t =-2.221, p = ns, [-19.42 - 2.13] | . |
|  | CT | t =0.556, p = ns, [-8.61 - 12.94] | t =-1.256, p = ns, [-15.66 - 5.89] | t =0.965, p = ns, [-7.02 - 14.53] |

*Holm corrected for 6 comparisons

**Supplementary Table 7**

Descriptive statistics of spontaneous eye blink rate (in blinks per minute) per location and block

|  | **Sham** | **C** | **T** | **CT** |
| --- | --- | --- | --- | --- |
|  | **mean (+/-sd)** | **mean (+/-sd)** | **mean (+/-sd)** | **mean (+/-sd)** |
| **Block #1, rest, tVNS off** | 11.97 (10.75) | 14.12 (11.28) | 13.21 (12.42) | 12.35 (10.75) |
| **Block #2, sipping, tVNS off** | 20.22 (10.42) | 18.26 (11.63) | 18.44 (10.77) | 20.33 (12.08) |
| **Block #3, rest, tVNS off** | 18.87 (11.94) | 16.56 (14.4) | 13.12 (10.3) | 16.28 (12.6) |
| **Block #4, rest, tVNS on** | 17.97 (14.19) | 16.95 (10.82) | 17.75 (13.43) | 17.16 (15.21) |
| **Block #5, sipping, tVNS on** | 21.41 (11.76) | 18.15 (13.4) | 18.72 (11.05) | 20.16 (10.35) |
| **Block #6, rest, tVNS on** | 18.64 (14.01) | 18.05 (12.25) | 16.28 (14.13) | 17.56 (14.21) |
| **Block #7 rest, tVNS off** | 17.65 (12.69) | 17.8 (13.22) | 15.61 (10.66) | 15.65 (12.1) |

**Supplementary Table 8**

Statistics of planned comparison p-values for t-tests of spontaneous eye-blink rate (in blinks per minute) between tVNS locations

| **Location** | **t-value, p-value*, [95% CI]** | | |
| --- | --- | --- | --- |
|  | **vs sham** | **vs C** | **vs T** |
| **Sham** |  |  |  |
| **C** | t = 0.838, p = 1, [-2.251 - 4.205] | . | . |
| **T** | t = 1.667, p = 0.618, [-1.285 - 5.171] | t = 0.828, p = 1, [-2.262 - 4.193] | . |
| **CT** | t = 0.888, p = 1, [-2.193 - 4.263] | t = 0.05, p = 1, [-3.17 - 3.286] | t = -0.779, p = 1, [-4.135 - 2.32] |

* Holm corrected for 6 estimates

**Supplementary Table 9**

Statistics of planned comparison p-values for t-tests of spontaneous eye-blink rate between blocks

| **Block** | **t-value, p-value*, [95% CI]** | | | | | |
| --- | --- | --- | --- | --- | --- | --- |
|  | **Block #1** | **Block #2** | **Block #3** | **Block #4** | **Block #5** | **Block #6** |
| **Vs Block #2** | **t = -5.205, p = < .001, [-10.25 - -2.547]** |  |  |  |  |  |
| **Vs Block #3** | t = -2.679, p = 0.133, [-7.145 - 0.558] | t = 2.526, p = 0.188, [-0.747 - 6.957] |  |  |  |  |
| **Vs Block #4** | **t = -3.696, p = 0.007, [-8.395 - -0.692]** | t = 1.509, p = 1, [-1.997 - 5.707] | t = -1.017, p = 1, [-5.102 - 2.602] |  |  |  |
| **Vs Block #5** | **t = -5.448, p = < .001, [-10.548 - -2.845]** | t = -0.243, p = 1, [-4.15 - 3.553] | t = -2.769, p = 0.111, [-7.255 - 0.448] | t = -1.752, p = 0.918, [-6.005 - 1.698] |  |  |
| **Vs Block #6** | **t = -3.84, p = 0.005, [-8.572 - -0.868]** | t = 1.365, p = 1, [-2.173 - 5.53] | t = -1.161, p = 1, [-5.278 - 2.425] | t = -0.144, p = 1, [-4.028 - 3.675] | t = 1.608, p = 1, [-1.875 - 5.828] |  |
| **Vs Block #7** | t = -3.064, p = 0.05, [-7.618 - 0.085] | t = 2.141, p = 0.422, [-1.22 - 6.483] | t = -0.385, p = 1, [-4.325 - 3.378] | t = 0.632, p = 1, [-3.075 - 4.628] | t = 2.384, p = 0.252, [-0.922 - 6.782] | t = 0.776, p = 1, [-2.898 - 4.805] |

* Holm corrected for 21 estimates

**Supplementary Table 10**

Statistics of ANOVA of internal and other state ratings

| **Scale** | **Effect of time** | **Effect of location** | **Interaction time*location** |
| --- | --- | --- | --- |
| **Comfort** | F(2,26)=5.119, p=0.013, p_FDR_ = ns*, η_p_^2^=0.283 | F(3,39)=1.475, p=0.236, η_p_^2^²=0.102 | F(6,78)=0.576, p=0.749, η_p_^2^=0.042 |
| **Fullness** | F(2,26)=1.114, p=0.343, η_p_^2^=0.079 | F(3,39)=0.707, p=0.553, η_p_^2^=0.052 | F(6,78)=1.194, p=0.318, η_p_^2^=0.084 |
| **Temperature** | F(2,26)=3.086, p=0.063, η_p_^2^=0.192 | F(3,39)=1.275, p=0.296, η_p_^2^=0.089 | F(6,78)=1.087, p=0.378, η_p_^2^=0.077 |
| **Hunger** | F(2,26)=3.74, p=0.037, p_FDR_ = ns*, η_p_^2^=0.223 | F(3,39)=0.629, p=0.601, η_p_^2^=0.046 | F(6,78)=0.808, p=0.567, η_p_^2^=0.059 |
| **Need to pee** | F(2,26)=2.809, p=0.079, η_p_^2^=0.178 | F(3,39)=0.349, p=0.79, η_p_^2^=0.026 | F(6,78)=1.138, p=0.348, η_p_^2^=0.081 |
| **Sleepiness** | F(2,26)=4.332, p=0.187, η_p_^2^=0.187 | F(3,39)=1.212, p=0.318, η_p_^2^=0.085 | F(6,78)=1.192, p=0.32, η_p_^2^=0.084 |
| **Stress** | F(2,26)=0.623, p=0.544, η_p_^2^=0.046 | F(3,39)=0.173, p=0.914, η_p_^2^=0.013 | F(6,78)=0.456, p=0.838, η_p_^2^=0.034 |
| **Thirst** | F(2,26)=6.242, p=0.006, p_FDR_ = ns*, η_p_^2^=0.324 | F(3,39)=0.572, p=0.637, η_p_^2^=0.042 | F(6,78)=0.922, p=0.484, η_p_^2^=0.066 |
| **Thirst (with session order covariate)** | F(2,24)=2.563, p=0.098, η_p_^2^=0.167 | location*session order:  F(3,36)=5.05, p=0.005, η_p_^2^=0.296  Location: F(3,36)=3.341, p=0.030, p_FDR_ = ns*, η_p_^2^=0.218 | F(6,72)=0.865, p=0.525, η_p_^2^=0.067 |
| **Tiredness** | **F(2,26)=10.202, p=< .001, p_FDR_ = 0.015, η_p_^2^=0.44** | F(3,39)=1.209, p=0.319, η_p_^2^=0.085 | F(6,78)=0.591, p=0.737, η_p_^2^=0.043 |

* corrected for false discovery rate with the Benjamini-Hochberg procedure for a family of 27 tests

**Supplementary Table 11**

Planned comparison p-values for t-tests for time on internal and other state ratings

| **VAS label** | **Time point** | **t-value, p-value*, [95% CI]** | |
| --- | --- | --- | --- |
|  |  | **vs Before block #1, tNVS off** | **vs Before block #4, tVNS on** |
| **Hunger** | Before block #1, tNVS off | . | . |
|  | Before block #4, tVNS on | **t =2.587, p = 0.047, [0.09 - 16.98]** | . |
|  | After block #7, tVNS off | t =2.062, p = ns, [-1.64 - 15.25] | t =-0.524, p = ns, [-10.17 - 6.71] |
| **Fullness** | Before block #1, tNVS off | . | . |
|  | Before block #4, tVNS on | t =-1.158, p = ns, [-14.38 - 5.42] | . |
|  | After block #7, tVNS off | t =-1.395, p = ns, [-15.3 - 4.5] | t =-0.238, p = ns, [-10.82 - 8.98] |
| **Thirst** | Before block #1, tNVS off | . | . |
|  | Before block #4, tVNS on | t =-0.278, p = ns, [-6.45 - 5.19] | . |
|  | After block #7, tVNS off | **t =-3.189, p = 0.011, [-13.07 - -1.43]** | **t =-2.911, p = 0.015, [-12.43 - -0.8]** |
| **Need to Pee** | Before block #1, tNVS off | . | . |
|  | Before block #4, tVNS on | t =-0.247, p = ns, [-7.04 - 5.8] | . |
|  | After block #7, tVNS off | t =-2.165, p = ns, [-11.86 - 0.99] | t =-1.918, p = ns, [-11.24 - 1.61] |
| **Tiredness** | Before block #1, tNVS off | . | . |
|  | Before block #4, tVNS on | t =-2.034, p = ns, [-12.06 - 1.38] | . |
|  | After block #7, tVNS off | t =-4.51, p = ns, [-18.56 - -5.12] | **t =-2.476, p = 0.04, [-13.22 - 0.22]** |
| **Sleepiness** | Before block #1, tNVS off | . | . |
|  | Before block #4, tVNS on | t =-2.255, p = ns, [-17.3 - 1.09] | . |
|  | After block #7, tVNS off | **t =-2.766, p = 0.031, [-19.14 - -0.75]** | t =-0.511, p = ns, [-11.04 - 7.36] |
| **Stress** | Before block #1, tNVS off | . | **.** |
|  | Before block #4, tVNS on | t =0.632, p = ns, [-4.74 - 7.85] | **.** |
|  | After block #7, tVNS off | t =1.113, p = ns, [-3.56 - 9.03] | t =0.48, p = ns, [-5.11 - 7.48] |
| **Comfort** | Before block #1, tNVS off | . | . |
|  | Before block #4, tVNS on | t =1.593, p = ns, [-3.28 - 14.11] | . |
|  | After block #7, tVNS off | **t =3.2, p = 0.011, [2.18 - 19.58]** | t =1.606, p = ns, [-3.24 - 14.16] |
| **Temperature** | Before block #1, tNVS off | . | . |
|  | Before block #4, tVNS on | t =-1.671, p = ns, [-5.77 - 1.21] | . |
|  | After block #7, tVNS off | t =-2.428, p = ns, [-6.8 - 0.18] | t =-0.756, p = ns, [-4.52 - 2.46] |

*Holm corrected for 3 comparisons

**Supplementary Table 12**

Planned comparison p-values for t-tests for location on internal and other state ratings

| **VAS label** | **Location** | **t-value, p-value*, [95% CI]** | | |
| --- | --- | --- | --- | --- |
|  |  | **vs sham*** | **vs C** | **vs T** |
| **Hunger** | Sham |  |  |  |
|  | C | t =0.399, p = ns, [-11.25 - 15.01] | . | . |
|  | T | t =-0.938, p = ns, [-17.56 - 8.7] | t =-1.336, p = ns, [-19.44 - 6.82] | . |
|  | CT | t =-0.122, p = ns, [-13.7 - 12.55] | t =-0.52, p = ns, [-15.59 - 10.67] | t =0.816, p = ns, [-9.27 - 16.98] |
| **Fullness** | Sham |  |  |  |
|  | C | t =-0.854, p = ns, [-17.88 - 9.47] | . | . |
|  | T | t =0.543, p = ns, [-11.01 - 16.35] | t =1.397, p = ns, [-6.8 - 20.55] | . |
|  | CT | t =0.199, p = ns, [-12.69 - 14.66] | t =1.054, p = ns, [-8.49 - 18.86] | t =-0.343, p = ns, [-15.36 - 11.99] |
| **Thirst** | Sham |  |  |  |
|  | C | t =-1.156, p = ns, [-20.99 - 8.66] | . | . |
|  | T | t =-0.089, p = ns, [-15.3 - 14.35] | t =1.067, p = ns, [-9.14 - 20.52] | . |
|  | CT | t =-0.216, p = ns, [-15.98 - 13.68] | t =0.941, p = ns, [-9.81 - 19.84] | t =-0.126, p = ns, [-15.5 - 14.15] |
| **Need to pee** | Sham |  |  |  |
|  | C | t =0.548, p = ns, [-8.58 - 12.8] | . | . |
|  | T | t =1.003, p = ns, [-6.83 - 14.54] | t =0.455, p = ns, [-8.94 - 12.44] | . |
|  | CT | t =0.355, p = ns, [-9.32 - 12.06] | t =-0.193, p = ns, [-11.43 - 9.95] | t =-0.647, p = ns, [-13.18 - 8.2] |
| **Tiredness** | Sham |  |  |  |
|  | C | t =-1.571, p = ns, [-18.33 - 5.09] | . | . |
|  | T | t =-0.129, p = ns, [-12.26 - 11.17] | t =1.442, p = ns, [-5.64 - 17.78] | . |
|  | CT | t =0.057, p = ns, [-11.47 - 11.95] | t =1.628, p = ns, [-4.85 - 18.57] | t =0.186, p = ns, [-10.93 - 12.5] |
| **Sleepiness** | Sham |  |  |  |
|  | C | t =-1.746, p = ns, [-22.22 - 5.08] | . | . |
|  | T | t =-0.79, p = ns, [-17.53 - 9.77] | t =0.956, p = ns, [-8.95 - 18.34] | . |
|  | CT | t =-0.224, p = ns, [-14.75 - 12.55] | t =1.522, p = ns, [-6.17 - 21.12] | t =0.566, p = ns, [-10.87 - 16.43] |
| **Stress** | Sham |  |  |  |
|  | C | t =-0.343, p = ns, [-13.38 - 10.44] | . | . |
|  | T | t =-0.707, p = ns, [-14.94 - 8.88] | t =-0.364, p = ns, [-13.47 - 10.35] | . |
|  | CT | t =-0.236, p = ns, [-12.92 - 10.89] | t =0.107, p = ns, [-11.45 - 12.37] | t =0.471, p = ns, [-9.89 - 13.92] |
| **Comfort** | Sham |  |  |  |
|  | C | t =0.786, p = ns, [-6.83 - 12.22] | . | . |
|  | T | t =1.35, p = ns, [-4.9 - 14.15] | t =0.564, p = ns, [-7.59 - 11.46] | . |
|  | CT | t =2.025, p = ns, [-2.59 - 16.46] | t =1.239, p = ns, [-5.28 - 13.77] | t =0.674, p = ns, [-7.21 - 11.83] |
| **Temperature** | Sham |  |  |  |
|  | C | t =-0.067, p = ns, [-4.13 - 3.94] | . | . |
|  | T | t =1.381, p = ns, [-2.03 - 6.04] | t =1.448, p = ns, [-1.93 - 6.14] | . |
|  | CT | t =-0.438, p = ns, [-4.67 - 3.4] | t =-0.371, p = ns, [-4.57 - 3.5] | t =-1.819, p = ns, [-6.68 - 1.39] |

*Holm corrected for 6 comparisons

**Supplementary Table 13**

Descriptive statistics of perceptual ratings of the palatable drink per location before recording

| **VAS label** | **Time** | **Sham** | **C** | **T** | **CT** |
| --- | --- | --- | --- | --- | --- |
|  |  | **mean (+/-sd)** | **mean (+/-sd)** | **mean (+/-sd)** | **mean (+/-sd)** |
| **Liking** | **Before block #1, tNVS off** | 30.86 (15.53) | 30.51 (13.85) | 31.6 (13.16) | 34.36 (14.15) |
|  | **Before block #4, tVNS on** | 31.15 (14.46) | 31.65 (15.22) | 27.95 (15.98) | 29.49 (14.66) |
|  | **After block #7, tVNS off** | 25.79 (22.02) | 27.91 (19.45) | 23.85 (20.92) | 25.46 (21.89) |
| **Wanting** | **Before block #1, tNVS off** | 77.43 (24.32) | 78.07 (24.79) | 74.85 (23.53) | 76.77 (24.09) |
|  | **Before block #4, tVNS on** | 73.6 (23.85) | 74.63 (24.8) | 72.13 (24.78) | 72.14 (22.83) |
|  | **After block #7, tVNS off** | 64.61 (33.47) | 65.14 (31.03) | 59.25 (32.04) | 60.37 (30.71) |
| **Intensity** | **Before block #1, tNVS off** | 65 (16.88) | 58.61 (22.4) | 62.25 (22.27) | 65.95 (12.73) |
|  | **Before block #4, tVNS on** | 66.52 (21.58) | 63.25 (15.64) | 64.91 (15.92) | 66.07 (18.63) |
|  | **After block #7, tVNS off** | 72.48 (13.8) | 66.05 (15.63) | 67.15 (18.37) | 71.55 (17.21) |
| **Sweetness** | **Before block #1, tNVS off** | 68.17 (16.28) | 60.75 (21.38) | 63.48 (16.04) | 73.84 (7.73) |
|  | **Before block #4, tVNS on** | 66.49 (14.92) | 66.85 (10.01) | 68.37 (12.71) | 72.62 (13) |
|  | **After block #7, tVNS off** | 75.12 (11.3) | 71.2 (11.08) | 69.75 (14.3) | 73.31 (12.68) |
| **Sourness** | **Before block #1, tNVS off** | 1.61 (4.61) | 1.26 (2.43) | 2.49 (5.15) | 1.67 (3.73) |
|  | **Before block #4, tVNS on** | 1.27 (4.73) | 1.05 (3.88) | 4.47 (7.64) | 2.11 (5.37) |
|  | **After block #7, tVNS off** | 1.1 (4.13) | 0.72 (2.67) | 1.42 (2.85) | 1.31 (4.84) |
| **Bitter** | **Before block #1, tNVS off** | 3.44 (5.88) | 1.35 (2.06) | 2.86 (4.14) | 0.87 (1.96) |
|  | **Before block #4, tVNS on** | 2.26 (4.18) | 2.04 (4.52) | 1.94 (2.11) | 1.34 (3.26) |
|  | **After block #7, tVNS off** | 1.54 (3.61) | 2.46 (3.51) | 4.98 (7.9) | 3.12 (5.97) |
| **Creamy** | **Before block #1, tNVS off** | 66.59 (20.91) | 65.13 (24.02) | 56.6 (26.02) | 66.95 (18.19) |
|  | **Before block #4, tVNS on** | 63.44 (24.58) | 58.59 (22.66) | 63.34 (25.03) | 63.63 (21.93) |
|  | **After block #7, tVNS off** | 67.09 (23.38) | 60.79 (22) | 64.14 (22.85) | 63.41 (23.14) |
| **Fatty** | **Before block #1, tNVS off** | 59.86 (23.79) | 60.19 (25.29) | 55.15 (24.75) | 64.49 (20.58) |
|  | **Before block #4, tVNS on** | 61.15 (27.31) | 58.28 (20.85) | 59.64 (20.93) | 64.07 (23.85) |
|  | **After block #7, tVNS off** | 65.66 (24.79) | 59.12 (23.04) | 63.18 (21.55) | 60.91 (23.46) |
| **Other** | **Before block #1, tNVS off** | 10.24 (24.23) | 12.47 (26.61) | 16.04 (31.46) | 15.71 (29.78) |
|  | **Before block #4, tVNS on** | 14.47 (29.65) | 10.31 (20.87) | 16.23 (30.53) | 11.6 (26.75) |
|  | **After block #7, tVNS off** | 13.36 (32.29) | 12.68 (27.57) | 12.33 (30.1) | 11.84 (28.03) |
| **Familiarity** | **Before block #1, tNVS off** | 39.34 (12.08) | 39.72 (11.5) | 38.95 (12.65) | 37.73 (14.47) |
|  | **Before block #4, tVNS on** | 37.95 (12.2) | 39.37 (10.97) | 37.18 (14.06) | 36.38 (13.39) |
|  | **After block #7, tVNS off** | 39.52 (11.17) | 40.52 (11.67) | 37.2 (13.87) | 36.57 (13.28) |
| **Calorie** | **Before block #1, tNVS off** | 70.16 (18.98) | 69.02 (18.89) | 67.15 (16.89) | 76.31 (13.42) |
|  | **Before block #4, tVNS on** | 70.53 (17.37) | 67.45 (13.01) | 68.36 (13.97) | 73.06 (15.41) |
|  | **After block #7, tVNS off** | 74.48 (11.25) | 72.67 (15.08) | 72.9 (14.69) | 75.38 (13.28) |
| **Healthy** | **Before block #1, tNVS off** | -10.06 (23.01) | -8.29 (22.64) | -12.7 (16.98) | -14.36 (21.56) |
|  | **Before block #4, tVNS on** | -9.63 (19.45) | -8.85 (22.63) | -10.66 (19.66) | -14.4 (22.91) |
|  | **After block #7, tVNS off** | -13.61 (21.7) | -12.37 (21.53) | -16.07 (22.21) | -16.06 (21.14) |

**Supplementary Table 14**

Statistics of ANOVA of perceptual ratings of the palatable drink per time and location

| **Scale** | **Effect of time** | **Effect of location** | **Interaction time*location** |
| --- | --- | --- | --- |
| **Liking** | F(1.127,14.648), =1.675, p = 0.218, η_p_^2^ = 0.114 | F(2.096,27.249), =0.95, p = 0.403, η_p_^2^ = 0.068 | F(3.26,42.375), =1.502, p = 0.225, η_p_^2^ = 0.104 |
| **Wanting** | F(1.069,13.897), =4.868, p = 0.043, p_FDR_ = ns*, η_p_^2^ = 0.272 | F(3,39), =1.894, p = 0.147, η_p_^2^ = 0.127 | F(6,78), =0.469, p = 0.829, η_p_^2^ = 0.035 |
| **Intensity** | F(1.197,15.562), =1.696, p = 0.215, η_p_^2^ = 0.115 | F(3,39), =1.91, p = 0.144, η_p_^2^ = 0.128 | F(6,78), =0.256, p = 0.955, η_p_^2^ = 0.019 |
| **Sweetness** | F(1.081,14.05), =1.638, p = 0.223, η_p_^2^ = 0.112 | F(3,39), =4.08, p = 0.013, p_FDR_ = ns*, η_p_^2^ = 0.239 | F(3.046,39.592), = 2.206, p = 0.102, η_p_^2^ = 0.145 |
| **Bitter** | F(1.173,15.243), =0.958, p = 0.358, η_p_^2^ = 0.069 | F(3,39), =1.367, p = 0.267, η_p_^2^ = 0.095 | F(2.762,35.907), =1.471, p = 0.24, η_p_^2^ = 0.102 |
| **Sour** | F(2,26), =1.813, p = 0.183, η_p_^2^ = 0.122 | F(1.766,22.953), =1.036, p = 0.363, η_p_^2^ = 0.074 | F(2.614,33.976), = 1.559, p = 0.221, η_p_^2^ = 0.107 |
| **Creamy** | F(2,26), =0.498, p = 0.613, η_p_^2^ = 0.037 | F(3,39), =1.12, p = 0.353, η_p_^2^ = 0.079 | F(6,78), =2.325, p = 0.041, p_FDR_ = ns*, η_p_^2^ = 0.152 |
| **Fatty** | F(2,26), =1.238, p = 0.307, η_p_^2^ = 0.087 | F(3,39), =1.234, p = 0.311, η_p_^2^ = 0.087 | F(2.433,31.623), = 1.736, p = 0.187, η_p_^2^ = 0.118 |
| **Other** | F(2,26), =0.239, p = 0.789, η_p_^2^ = 0.018 | F(3,39), =0.662, p = 0.581, η_p_^2^ = 0.048 | F(3.221,41.879), = 1.582, p = 0.206, η_p_^2^ = 0.108 |
| **Familiarity** | F(2,26), =1.045, p = 0.366, η_p_^2^ = 0.074 | F(3,39), =1.14, p = 0.345, η_p_^2^ = 0.081 | F(2.558,33.253), =0.4, p = 0.723, η_p_^2^ = 0.03 |
| **Calorie** | F(1.106,14.372), =1.114, p = 0.316, η_p_^2^ = 0.079 | F(1.661,21.593), =2.107, p = 0.152, η_p_^2^ = 0.139 | F(6,78), =0.549, p = 0.77, η_p_^2^ = 0.04 |
| **Calorie (with session order covariate)** | F(1.107,13.289), =1.627, p = 0.218, η_p_^2^ = 0.119 | F(1.587,19.042), =0.265, p = 0.719, η_p_^2^ = 0.022 | time*location*session order: F(6,72), =2.564, p = 0.026, η_p_^2^ = 0.176  time*location: F(6,72), =2.378, p = 0.038, p_FDR_ = ns*, η_p_^2^ = 0.165 |
| **Healthy** | F(1.359,17.665), =2.098, p = 0.162, η_p_^2^ = 0.139 | F(3,39), =2.165, p = 0.108, η_p_^2^ = 0.143 | F(2.711,35.245), = 0.387, p = 0.743, η_p_^2^ = 0.029 |

1. Greenhouse Geisser statistics because Mauchly's test of sphericity indicated that the assumption of sphericity was violated (p < .05).

* corrected for false discovery rate with the Benjamini-Hochberg procedure for a family of 36 tests

**Supplementary Table 15**

Statistics of planned comparison p-values for t-tests for effect of time on perceptual ratings of the palatable drink

| **VAS label** | **Time point** | **t-value, p-value*, [95% CI]** | |
| --- | --- | --- | --- |
|  |  | **vs Before block #1, tNVS off** | **vs Before block #4, tVNS on** |
| **Liking** | **Before block #1, tNVS off** | . | . |
|  | **Before block #4, tVNS on** | t = 0.52, p = 0.608, [-6.97 - 10.52] | . |
|  | **After block #7, tVNS off** | t = 1.78, p = 0.26, [-2.66 - 14.83] | t = 1.26, p = 0.437, [-4.44 - 13.05] |
| **Wanting** | **Before block #1, tNVS off** | . | . |
|  | **Before block #4, tVNS on** | t = 0.76, p = 0.454, [-8.65 - 15.97] | . |
|  | **After block #7, tVNS off** | **t = 3, p = 0.018, [2.13 - 26.74]** | t = 2.24, p = 0.068, [-1.53 - 23.09] |
| **Intensity** | **Before block #1, tNVS off** | . | . |
|  | **Before block #4, tVNS on** | t = -0.64, p = 0.529, [-11.19 - 6.72] | . |
|  | **After block #7, tVNS off** | t = -1.82, p = 0.243, [-15.31 - 2.6] | t = -1.18, p = 0.5, [-13.08 - 4.84] |
| **Sweetness** | **Before block #1, tNVS off** | . | . |
|  | **Before block #4, tVNS on** | t = -0.62, p = 0.538, [-10.33 - 6.28] | . |
|  | **After block #7, tVNS off** | t = -1.78, p = 0.259, [-14.09 - 2.52] | t = -1.16, p = 0.514, [-12.06 - 4.54] |
| **Sourness** | **Before block #1, tNVS off** | . | . |
|  | **Before block #4, tVNS on** | t = -0.82, p = 0.583, [-1.94 - 1] | . |
|  | **After block #7, tVNS off** | t = 1.08, p = 0.583, [-0.85 - 2.09] | t = 1.9, p = 0.206, [-0.38 - 2.56] |
| **Bitterness** | **Before block #1, tNVS off** | . | . |
|  | **Before block #4, tVNS on** | t = 0.27, p = 0.787, [-1.97 - 2.44] | . |
|  | **After block #7, tVNS off** | t = -1.04, p = 0.617, [-3.1 - 1.31] | t = -1.31, p = 0.603, [-3.34 - 1.08] |
| **Creaminess** | **Before block #1, tNVS off** | . | **.** |
|  | **Before block #4, tVNS on** | t = 0.85, p = 1, [-3.13 - 6.26] | **.** |
|  | **After block #7, tVNS off** | t = -0.02, p = 1, [-4.73 - 4.65] | t = -0.88, p = 1, [-6.3 - 3.09] |
| **Fattiness** | **Before block #1, tNVS off** | . | . |
|  | **Before block #4, tVNS on** | t = -0.58, p = 0.679, [-4.64 - 2.92] | . |
|  | **After block #7, tVNS off** | t = -1.56, p = 0.394, [-6.08 - 1.48] | t = -0.97, p = 0.679, [-5.21 - 2.34] |
| **Other** | **Before block #1, tNVS off** | . | . |
|  | **Before block #4, tVNS on** | t = 0.3, p = 1, [-3.49 - 4.43] | . |
|  | **After block #7, tVNS off** | t = 0.69, p = 1, [-2.89 - 5.03] | t = 0.39, p = 1, [-3.36 - 4.56] |
| **Familiarity** | **Before block #1, tNVS off** | . | . |
|  | **Before block #4, tVNS on** | t = 1.44, p = 0.489, [-0.95 - 3.38] | . |
|  | **After block #7, tVNS off** | t = 0.57, p = 0.791, [-1.68 - 2.65] | t = -0.86, p = 0.791, [-2.9 - 1.44] |
| **Calorie** | **Before block #1, tNVS off** | . | . |
|  | **Before block #4, tVNS on** | t = 0.29, p = 0.777, [-6.45 - 8.08] | . |
|  | **After block #7, tVNS off** | t = -1.13, p = 0.541, [-10.46 - 4.07] | t = -1.41, p = 0.509, [-11.27 - 3.26] |
| **Healthiness** | **Before block #1, tNVS off** | . | . |
|  | **Before block #4, tVNS on** | t = -0.24, p = 0.812, [-5.42 - 4.49] | . |
|  | **After block #7, tVNS off** | t = 1.64, p = 0.225, [-1.78 - 8.13] | t = 1.88, p = 0.213, [-1.31 - 8.6] |

*Holm corrected for 3 comparisons

**Supplementary Table 16**

Statistics of planned comparison p-values for t-tests for effect of location on perceptual ratings of the palatable drink

| **VAS label** | **Location** | **t-value, p-value*, [95% CI]** | | |
| --- | --- | --- | --- | --- |
|  |  | **vs sham** | **vs C** | **vs T** |
| **Liking** | Sham | . | . | . |
|  | C | t = -0.53, p = 1, [-4.77 - 3.25] | . | . |
|  | T | t = 1.02, p = 1, [-2.54 - 5.47] | t = 1.54, p = 0.788, [-1.79 - 6.23] | . |
|  | CT | t = -0.35, p = 1, [-4.51 - 3.5] | t = 0.18, p = 1, [-3.75 - 4.26] | t = -1.37, p = 0.9, [-5.98 - 2.04] |
| **Wanting** | Sham | . | . | . |
|  | C | t = -0.4, p = 1, [-5.89 - 4.41] | . | . |
|  | T | t = 1.69, p = 0.493, [-2.01 - 8.29] | t = 2.09, p = 0.259, [-1.28 - 9.02] | . |
|  | CT | t = 1.14, p = 0.781, [-3.03 - 7.27] | t = 1.54, p = 0.527, [-2.3 - 8] | t = -0.55, p = 1, [-6.17 - 4.13] |
| **Intensity** | Sham | . | . | . |
|  | C | t  = 2.02, p = 0.3, [-2.01 - 12.72] | . | . |
|  | T | t = 1.22, p = 0.922, [-4.14 - 10.59] | t = -0.81, p = 0.922, [-9.5 - 5.23] | . |
|  | CT | t = 0.05, p = 0.958, [-7.23 - 7.5] | t = -1.97, p = 0.3, [-12.58 - 2.15] | t = -1.17, p = 0.922, [-10.45 - 4.28] |
| **Sweetness** | Sham | . | . | . |
|  | C | t  = 1.67, p = 0.415, [-2.45 - 9.77] | . | . |
|  | T | t = 1.24, p = 0.444, [-3.38 - 8.84] | t = -0.43, p = 0.673, [-7.05 - 5.18] | . |
|  | CT | t = -1.51, p = 0.415, [-9.44 - 2.78] | **t = -3.18, p = 0.017, [-13.1 - -0.88]** | **t = -2.75, p = 0.044, [-12.17 - 0.06]** |
| **Sourness** | Sham | . | . | . |
|  | C | t = 0.29, p = 1, [-2.69 - 3.32] | . | . |
|  | T | t = -1.36, p = 0.91, [-4.47 - 1.54] | t = -1.65, p = 0.639, [-4.79 - 1.22] | . |
|  | CT | t = -0.35, p = 1, [-3.38 - 2.63] | t = -0.64, p = 1, [-3.69 - 2.31] | t = 1.01, p = 1, [-1.91 - 4.1] |
| **Bitter** | Sham | . | . | . |
|  | C | t = 0.58, p = 1, [-1.77 - 2.7] | . | . |
|  | T | t = -1.06, p = 1, [-3.08 - 1.39] | t = -1.63, p = 0.556, [-3.54 - 0.92] | . |
|  | CT | t = 0.8, p = 1, [-1.6 - 2.87] | t = 0.22, p = 1, [-2.06 - 2.41] | t = 1.85, p = 0.431, [-0.75 - 3.72] |
| **Creamy** | Sham | . | . | . |
|  | C | t  = 1.42, p = 0.894, [-4 - 12.41] | . | . |
|  | T | t = 1.47, p = 0.894, [-3.86 - 12.55] | t = 0.05, p = 1, [-8.07 - 8.35] | . |
|  | CT | t = 0.36, p = 1, [-7.16 - 9.25] | t = -1.07, p = 1, [-11.37 - 5.05] | t = -1.12, p = 1, [-11.51 - 4.91] |
| **Fatty** | Sham | . | . | . |
|  | C | t = 1.18, p = 0.983, [-4.11 - 10.16] | . | . |
|  | T | t = 1.13, p = 0.983, [-4.24 - 10.04] | t = -0.05, p = 1, [-7.26 - 7.01] | . |
|  | CT | t = -0.36, p = 1, [-8.07 - 6.21] | t = -1.54, p = 0.788, [-11.1 - 3.18] | t = -1.49, p = 0.788, [-10.97 - 3.31] |
| **Other** | Sham | . | . | . |
|  | C | t = 0.39, p = 1, [-5.32 - 7.06] | . | . |
|  | T | t = -0.98, p = 1, [-8.37 - 4.02] | t = -1.37, p = 1, [-9.24 - 3.15] | . |
|  | CT | t = -0.16, p = 1, [-6.55 - 5.83] | t = -0.55, p = 1, [-7.42 - 4.96] | t = 0.81, p = 1, [-4.38 - 8.01] |
| **Familiarity** | Sham | . | . | . |
|  | C | t = -0.54, p = 1, [-5.73 - 3.87] | . | . |
|  | T | t = 0.67, p = 1, [-3.64 - 5.96] | t = 1.21, p = 1, [-2.71 - 6.89] | . |
|  | CT | t = 1.18, p = 1, [-2.76 - 6.85] | t = 1.72, p = 0.556, [-1.82 - 7.78] | t = 0.51, p = 1, [-3.91 - 5.69] |
| **Calorie** | Sham | . | . | . |
|  | C | t = 0.82, p = 1, [-4.81 - 8.83] | . | . |
|  | T | t = 0.92, p = 1, [-4.56 - 9.08] | t = 0.1, p = 1, [-6.58 - 7.07] | . |
|  | CT | t = -1.3, p = 0.805, [-10.01 - 3.63] | t = -2.12, p = 0.202, [-12.02 - 1.62] | t = -2.22, p = 0.194, [-12.27 - 1.37] |
| **Healthy** | Sham | . | . | . |
|  | C | t = -0.59, p = 1, [-7.28 - 4.74] | . | . |
|  | T | t = 0.94, p = 1, [-3.97 - 8.05] | t = 1.53, p = 0.537, [-2.7 - 9.32] | . |
|  | CT | t = 1.78, p = 0.418, [-2.17 - 9.85] | t = 2.36, p = 0.14, [-0.91 - 11.11] | t = 0.83, p = 1, [-4.21 - 7.81] |

*Holm corrected for 6 comparisons

**Supplementary Table 17**

Descriptive statistics of amount of palatable drink consumed (in g) per cycle per stimulation location during block #2, sipping, tVNS off

|  | **Sham** | **C** | **T** | **CT** |
| --- | --- | --- | --- | --- |
| **Cycle** | **mean (+/-sd)** | **mean (+/-sd)** | **mean (+/-sd)** | **mean (+/-sd)** |
| **1** | 6.68 (4.13) | 7.27 (5.4) | 4.28 (3.48) | 5.36 (3.58) |
| **2** | 8.62 (7.14) | 8.59 (6.9) | 6.86 (5.39) | 6.69 (4.23) |
| **3** | 7.62 (5.56) | 9.32 (7.67) | 5.54 (3.45) | 7.88 (4.82) |
| **4** | 7.46 (5.43) | 9.69 (7.18) | 6.52 (6.05) | 7.16 (4.2) |
| **5** | 8.53 (6.46) | 10.14 (6.63) | 6.77 (5.35) | 7.08 (3.88) |
| **6** | 7.81 (5.35) | 8.84 (5.27) | 6.19 (3.15) | 7.06 (3.88) |
| **7** | 7.58 (5.89) | 8.06 (4.1) | 7.32 (5.7) | 6.7 (3.32) |
| **8** | 7.22 (5.84) | 8.5 (4.96) | 5.94 (3.1) | 6.38 (3.04) |
| **9** | 8.4 (5.86) | 4.7 (14.37) | 6.25 (3.51) | 6.62 (3.45) |
| **10** | 7.26 (5.43) | 8.79 (5.05) | 6.39 (3.5) | 6.7 (3.46) |

**Supplementary Table 18**

Descriptive statistics of amount of palatable drink consumed (in g) per cycle per stimulation location during block #5, sipping, tVNS on

|  | **Sham** | **C** | **T** | **CT** |
| --- | --- | --- | --- | --- |
| **Cycle** | **mean (+/-sd)** | **mean (+/-sd)** | **mean (+/-sd)** | **mean (+/-sd)** |
| **1** | 6.71 (4.76) | 5.82 (4.26) | 6.59 (5.96) | 5.65 (2.75) |
| **2** | 8.04 (6.22) | 8.48 (4.6) | 6.6 (3.59) | 7.68 (4.55) |
| **3** | 7.58 (6.38) | 7.86 (4.2) | 6.65 (3.43) | 8.12 (4.1) |
| **4** | 7.87 (5.08) | 7.23 (5.4) | 8.36 (4.43) | 7.07 (3.2) |
| **5** | 7.23 (5.07) | 7.76 (4.36) | 6.77 (6.92) | 6.85 (3.71) |
| **6** | 7.94 (5.21) | 7.56 (5.26) | 8.34 (4.46) | 6.94 (2.82) |
| **7** | 7.32 (4.73) | 7.42 (4.74) | 8.26 (5.56) | 8.14 (4.03) |
| **8** | 7.61 (3.89) | 7.74 (4.43) | 7.74 (3.82) | 7.6 (3.89) |
| **9** | 7.11 (4.51) | 6.83 (5.04) | 8.15 (4.56) | 7.21 (3.04) |
| **10** | 6.31 (3.1) | 8.09 (4.92) | 8.7 (4.06) | 7.71 (3.77) |

**Supplementary Table 19**

Descriptive statistics of EMG swallowing measures peak amplitude and peak latency per stimulation location during block #2, sipping, tVNS off and block #5, sipping, tVNS on

|  |  | **Sham** | **C** | **T** | **CT** |
| --- | --- | --- | --- | --- | --- |
| **EMG measure** | **Time** | **mean (+/-sd)** | **mean (+/-sd)** | **mean (+/-sd)** | **mean (+/-sd)** |
| **Peak amplitude (in μV)** | **block #2, sipping, tVNS off** | 0.017 (0.008) | 0.015 (0.011) | 0.016 (0.008) | 0.016 (0.009) |
|  | **block #2, sipping, tVNS off** | 0.016 (0.009) | 0.016 (0.012) | 0.018 (0.008) | 0.016 (0.009) |
| **Peak latency (in ms)** | **block #5, sipping, tVNS on** | 1335.9 (509.1) | 1426.5 (707.5) | 1441.3 (321.3) | 1557.5 (640.1) |
|  | **block #5, sipping, tVNS on** | 1407 (412.8) | 1398.5 (478.8) | 1391.4 (390) | 1459.9 (417.3) |

**Supplementary Table 20**

Statistical results for the deviations from zero in ERP signals in block #2, sipping, tVNS off and block #5, sipping, tVNS on

|  | **Block** | **Corrected p-value, upper and lower bound values** | **Electrode** | **Timepoint** |
| --- | --- | --- | --- | --- |
| **sham** | **block #2, sipping, tVNS off** | 0.0448 - 0.0068 | F4 | 513 - 521 ms |
| **C** | **block #2, sipping, tVNS off** | 0.0496 - 0.0172 | P4 | 519 - 586 ms |
| **T** | **block #2, sipping, tVNS off** | 0.0476 - 0.0152 | Oz, AF4  F4 | 450 - 456 ms  463 - 474 ms |
| **CT** | **block #2, sipping, tVNS off** | 0.0476 - 0.0200 | Oz  P8 | 501 - 525 ms  526 ms. |
| **sham** | **block #5, sipping, tVNS on** | 0.0468 - 0.0028 | F3  Pz | 448 - 452 ms  1035 - 1066 ms |
| **C** | **block #5, sipping, tVNS on** | 0.2936 | NA | NA |
| **T** | **block #5, sipping, tVNS on** | 0.0496 - 0.0056 | AF7  Oz, AF3, POz  Oz, POz  Oz, F4, AF3, POz  Oz, F4, POz  Oz, P8, F4, POz  F3, P8, F4, POz  F3, P8, POz  P8, POz  AF3  AF7, AF3  P8, AF7, AF3  P8, AF7, AF3, POz  AF7, AF3, POz  AF7, AF3  AF3  P8 | 464 - 467  476 - 477 ms  478 - 479 ms  480 ms  481 ms  482 ms  483 ms  484 - 487 ms  488 - 489 ms  494 - 498 ms  499 - 503 ms  504 - 507 ms.  508 - 513 ms  514 - 517 ms  518 -520 ms  521 ms  542 - 548 ms |
| **CT** | **block #5, sipping, tVNS on** | 0.2152 | NA | NA |

**Supplementary Table 21**

Statistical results for the differences between stimulation locations in block #5, sipping, tVNS on

|  | **Corrected p-value, lower bound value** | **Electrode** | **Timepoint** |
| --- | --- | --- | --- |
| **sham vs C** | 0.4660 | NA | NA |
| **sham vs T** | 0.0672 | F8 | 201-202 ms |
| **sham vs CT** | 0.7116 | NA | NA |
| **C vs T** | 0.8968 | NA | NA |
| **C vs CT** | 0.2044 | NA | NA |
| **T vs CT** | 0.8768 | NA | NA |
